# Supplementary material for: Cascades of effectiveness of next-generation insecticide-treated nets against malaria, from entomological trials to real-life conditions
Source: Nat Commun. 2025 Dec 16;16:11162. doi: 10.1038/s41467-025-66130-y (PMC12708653; doi:10.1038/s41467-025-66130-y)
Supplement: Supplementary file 1 — Supplementary Information [file 41467_2025_66130_MOESM1_ESM.pdf]

Cascades of effectiveness of next-generation insecticide-treated nets against malaria, from entomological trials to real-life conditions

Supplementary material

Clara Champagne[1,2 *], Jeanne Lemant[1,2], Alphonce Assenga[3], Ummi A. Kibondo[3], Ruth G. Lekundayo[3], Emmanuel Mbuba[1,2,3], Jason Moore[1,2,3], Joseph B. Muganga[3], Watson S. Ntabaliba[3], Olukayode G. Odufuwa[1,2,3], Johnson Kyeba Swai[1,2,3], Maria Alexa[1,2], Roland Goers[1,2], Monica Golumbeanu[1,2], Nakul Chitnis[1,2], Amanda Ross[1,2], Raphael N'Guessan[4,5], Sarah Moore[1,2,3], Emilie Pothin[1,2,6]

1. Swiss Tropical and Public Health Institute, Basel Switzerland
2. University of Basel, Basel Switzerland
3. Ifakara Health Institute, Bagamoyo, Tanzania
4. London School of Hygiene and Tropical Medicine, Department of Disease Control, London, UK
5. Vector Control Product Evaluation Centre, Institut Pierre Richet, Bouaké, Côte d'Ivoire
6. Clinton Health Access Initiative, Boston, USA

*clara.champagne@swisstph.ch

Contents

1. Estimates of entomological efficacy for each EHT 2
2. Sample sizes and characteristics of all EHT data 6
3. Differences in vectorial capacity reduction between EHT arms 10
4. ITN effectiveness cascades for each EHT independently 10
5. Sensitivity analysis on cascade order 12
6. Description of the unpublished experimental hut trials 14
7. Estimates of entomological indicators using 24h holding time 16
8. MCMC convergence diagnostics 17
9. Calibration methodology for RCT simulation 27
10. Estimating ITN attrition and intervention usage from RCT surveys on ITN usage 28
11. Activity patterns 29

References 30

1. Estimates of entomological efficacy for each EHT

Reduction in host availability			
**Net type**	**EHT**	**Unwashed**	**Washed 20x/Aged**
Interceptor G2	Assenga et al. 2025 (Tanzania)	0.54 (0.39-0.67)	0.33 (0.17-0.46)
	Kibondo et al. 2022	0.69 (0.52-0.81)	0.69 (0.57-0.78)
	BIT080	0.45 (0.22-0.63)	0.34 (0.18-0.48)
	Martin et al. 2024 (*An. gambiae*)	0.18 (0.01-0.48)	0.62 (0.28-0.85)
	Martin et al. 2024 (*An. funestus*)	0.4 (0.04-0.77)	0.47 (0.08-0.79)
	Assenga et al. 2025 (C. d'Ivoire)	0.46 (0.33-0.58)	0.06 (0-0.17)
	Nguessan et al. 2016	0.51 (0.41-0.59)	0.44 (0.34-0.52)
	Sovegnon et al. 2024	0.26 (0.01-0.57)	0.36 (0.04-0.67)
Olyset Plus	Assenga et al. 2025 (Tanzania)	0.81 (0.7-0.89)	0.75 (0.65-0.83)
	BIT055	0.89 (0.84-0.92)	0.79 (0.73-0.83)
	Odufuwa et al. 2024	0.86 (0.8-0.9)	0.83 (0.78-0.87)
	Martin et al. 2024 (*An. gambiae*)	0.53 (0.14-0.81)	0.47 (0.13-0.73)
	Martin et al. 2024 (*An. funestus*)	0.48 (0.09-0.78)	0.42 (0.06-0.72)
	Assenga et al. 2025 (C. d'Ivoire)	0.81 (0.75-0.86)	0.48 (0.39-0.56)
Pyrethroid-only	Assenga et al. 2025 (Tanzania)	0.82 (0.75-0.88)	0.75 (0.68-0.82)
	Kibondo et al. 2022	0.78 (0.64-0.87)	0.76 (0.67-0.83)
	BIT080	0.49 (0.39-0.59)	0.34 (0.2-0.45)
	BIT055	0.78 (0.72-0.83)	0.71 (0.65-0.77)
	Odufuwa et al. 2024	0.59 (0.49-0.69)	0.64 (0.55-0.71)
	Martin et al. 2024 (*An. gambiae*)	0.44 (0.09-0.69)	0.27 (0.02-0.6)
	Martin et al. 2024 (*An. funestus*)	0.33 (0.02-0.68)	0.62 (0.25-0.87)
	Assenga et al. 2025 (C. d'Ivoire)	0.48 (0.38-0.57)	0.61 (0.53-0.68)
	Nguessan et al. 2016	0.67 (0.6-0.73)	0.53 (0.46-0.6)
	Sovegnon et al. 2024	0.07 (0-0.22)	0.11 (0-0.31)

Supplementary Table 1.1. *Numerical estimates of reduction in host availability for unwashed and washed/aged nets (posterior mean and 95% credible intervals), compared to untreated nets as control. Assenga et al.[3], Kibondo et al. [4], BIT055, BIT080[5], Odufuwa et al.[6], Martin et al.[7], N'Guessan et al.[8] and Sovegnon et al.[9] Holding time is 24 hours for BIT055 and Odufuwa et al.[6] and 72 hours for all other trials.*

Pre-prandial killing effect			
**Net type**	**EHT**	**Unwashed**	**Washed 20x/Aged**
Interceptor G2	Assenga et al. 2025 (Tanzania)	0.45 (0.41-0.48)	0.3 (0.27-0.32)
	Kibondo et al. 2022	0.43 (0.39-0.48)	0.41 (0.38-0.45)
	BIT080	0.44 (0.41-0.47)	0.34 (0.32-0.36)
	Martin et al. 2024 (*An. gambiae*)	0.42 (0.3-0.55)	0.05 (0-0.15)
	Martin et al. 2024 (*An. funestus*)	0.44 (0.27-0.61)	0.1 (0.01-0.25)
	Assenga et al. 2025 (C. d'Ivoire)	0.64 (0.59-0.68)	0.57 (0.54-0.61)
	Nguessan et al. 2016	0.54 (0.5-0.58)	0.45 (0.42-0.49)
	Sovegnon et al. 2024	0.63 (0.49-0.75)	0.47 (0.28-0.64)
Olyset Plus	Assenga et al. 2025 (Tanzania)	0.34 (0.31-0.37)	0.32 (0.29-0.35)
	BIT055	0.45 (0.42-0.47)	0.3 (0.28-0.33)
	Odufuwa et al. 2024	0.37 (0.35-0.39)	0.23 (0.21-0.26)
	Martin et al. 2024 (*An. gambiae*)	0.47 (0.34-0.59)	0.18 (0.09-0.3)
	Martin et al. 2024 (*An. funestus*)	0.32 (0.19-0.46)	0.05 (0-0.16)
	Assenga et al. 2025 (C. d'Ivoire)	0.31 (0.27-0.36)	0.18 (0.15-0.21)
Pyrethroid-only	Assenga et al. 2025	0.36 (0.34-0.38)	0.28 (0.26-0.31)
	Kibondo et al. 2022	0.25 (0.2-0.3)	0.25 (0.21-0.28)
	BIT080	0.27 (0.26-0.29)	0.25 (0.24-0.27)
	BIT055	0.35 (0.33-0.38)	0.27 (0.24-0.29)
	Odufuwa et al. 2024	0.19 (0.17-0.22)	0.19 (0.17-0.21)
	Martin et al. 2024 (*An. gambiae*)	0.05 (0-0.14)	0.11 (0.01-0.24)
	Martin et al. 2024 (*An. funestus*)	0.1 (0-0.24)	0.05 (0.01-0.18)
	Assenga et al. 2025 (C. d'Ivoire)	0.2 (0.16-0.24)	0.2 (0.17-0.24)
	Nguessan et al. 2016	0.16 (0.13-0.2)	0.09 (0.06-0.12)
	Sovegnon et al. 2024	0.24 (0.07-0.38)	0.39 (0.25-0.51)

Supplementary Table 1.2. *Numerical estimates of pre-prandial killing effect for unwashed and washed/aged nets (posterior mean and 95% credible intervals), compared to untreated nets as control. Assenga et al.[3], Kibondo et al. [4], BIT055, BIT080[5], Odufuwa et al.[6], Martin et al.[7], N'Guessan et al.[8] and Sovegnon et al.[9] Holding time is 24 hours for BIT055 and Odufuwa et al.[6] and 72 hours for all other trials.*

Post-prandial killing effect			
**Net type**	**EHT**	**Unwashed**	**Washed 20x/Aged**
Interceptor G2	Assenga et al. 2025 (Tanzania)	0.14 (0.05-0.26)	0.25 (0.18-0.34)
	Kibondo et al. 2022	0.52 (0.3-0.72)	0.43 (0.29-0.58)
	BIT080	0.18 (0.07-0.32)	0.35 (0.26-0.45)
	Martin et al. 2024 (*An. gambiae*)	0.42 (0.19-0.66)	0.2 (0.01-0.51)
	Martin et al. 2024 (*An. funestus*)	0.48 (0.15-0.8)	0.11 (0-0.36)
	Assenga et al. 2025 (C. d'Ivoire)	0.76 (0.66-0.84)	0.56 (0.49-0.62)
	Nguessan et al. 2016	0.47 (0.4-0.55)	0.47 (0.4-0.53)
	Sovegnon et al. 2024	0.6 (0.36-0.81)	0.47 (0.2-0.72)
Olyset Plus	Assenga et al. 2025 (Tanzania)	0.23 (0.07-0.44)	0.27 (0.14-0.43)
	BIT055	0.58 (0.41-0.75)	0.32 (0.22-0.43)
	Odufuwa et al. 2024	0.41 (0.25-0.56)	0.31 (0.19-0.43)
	Martin et al. 2024 (*An. gambiae*)	0.42 (0.11-0.74)	0.08 (0-0.26)
	Martin et al. 2024 (*An. funestus*)	0.18 (0.01-0.48)	0.08 (0-0.26)
	Assenga et al. 2025 (C. d'Ivoire)	0.52 (0.38-0.65)	0.28 (0.22-0.34)
Pyrethroid-only	Assenga et al. 2025 (Tanzania)	0.16 (0.06-0.3)	0.06 (0.01-0.13)
	Kibondo et al. 2022	0.25 (0.05-0.49)	0.15 (0.03-0.28)
	BIT080	0.15 (0.09-0.22)	0.34 (0.27-0.42)
	BIT055	0.24 (0.13-0.36)	0.18 (0.1-0.27)
	Odufuwa et al. 2024	0.04 (0-0.1)	0.2 (0.12-0.28)
	Martin et al. 2024 (*An. gambiae*)	0.06 (0-0.22)	0.08 (0-0.26)
	Martin et al. 2024 (*An. funestus*)	0.16 (0.01-0.43)	0.13 (0-0.41)
	Assenga et al. 2025 (C. d'Ivoire)	0.31 (0.23-0.38)	0.19 (0.13-0.26)
	Nguessan et al. 2016	0.06 (0.03-0.11)	0.05 (0.03-0.08)
	Sovegnon et al. 2024	0.05 (0-0.15)	0.07 (0-0.18)

Supplementary Table 1.3. *Numerical estimates of post-prandial killing effect for unwashed and washed/aged nets (posterior mean and 95% credible intervals), compared to untreated nets as control. Assenga et al.[3], Kibondo et al. [4], BIT055, BIT080[5], Odufuwa et al.[6], Martin et al.[7], N'Guessan et al.[8] and Sovegnon et al.[9] Holding time is 24 hours for BIT055 and Odufuwa et al.[6] and 72 hours for all other trials.*

Entomological efficacy (measured in terms of reduction in vectorial capacity)			
**Net type**	**EHT**	**Unwashed**	**Washed 20x/Aged**
Interceptor G2	Assenga et al. 2025 (Tanzania)	0.91 (0.9-0.93)	0.89 (0.87-0.91)
	Kibondo et al. 2022	0.94 (0.92-0.95)	0.93 (0.92-0.94)
	BIT080	0.92 (0.9-0.94)	0.92 (0.91-0.94)
	Martin et al. 2024 (*An. gambiae*)	0.95 (0.91-0.97)	0.8 (0.6-0.9)
	Martin et al. 2024 (*An. funestus*)	0.95 (0.89-0.98)	0.63 (0.31-0.83)
	Assenga et al. 2025 (C. d'Ivoire)	0.97 (0.97-0.98)	0.98 (0.97-0.98)
	Nguessan et al. 2016	0.96 (0.95-0.96)	0.95 (0.94-0.96)
	Sovegnon et al. 2024	0.98 (0.96-0.99)	0.95 (0.91-0.98)
Olyset Plus	Assenga et al. 2025 (Tanzania)	0.91 (0.89-0.92)	0.91 (0.89-0.92)
	BIT055	0.93 (0.92-0.93)	0.91 (0.9-0.92)
	Odufuwa et al. 2024	0.92 (0.91-0.93)	0.9 (0.89-0.91)
	Martin et al. 2024 (*An. gambiae*)	0.94 (0.91-0.97)	0.78 (0.61-0.88)
	Martin et al. 2024 (*An. funestus*)	0.88 (0.79-0.94)	0.63 (0.31-0.83)
	Assenga et al. 2025 (C. d'Ivoire)	0.92 (0.91-0.93)	0.86 (0.83-0.88)
Pyrethroid-only	Assenga et al. 2025 (Tanzania)	0.91 (0.9-0.91)	0.88 (0.86-0.89)
	Kibondo et al. 2022	0.9 (0.86-0.92)	0.88 (0.86-0.9)
	BIT080	0.86 (0.84-0.88)	0.9 (0.88-0.91)
	BIT055	0.91 (0.9-0.92)	0.89 (0.87-0.9)
	Odufuwa et al. 2024	0.81 (0.77-0.84)	0.86 (0.84-0.88)
	Martin et al. 2024 (*An. gambiae*)	0.63 (0.35-0.81)	0.64 (0.34-0.84)
	Martin et al. 2024 (*An. funestus*)	0.71 (0.39-0.89)	0.77 (0.52-0.89)
	Assenga et al. 2025 (C. d'Ivoire)	0.87 (0.85-0.9)	0.86 (0.83-0.88)
	Nguessan et al. 2016	0.82 (0.79-0.85)	0.72 (0.67-0.76)
	Sovegnon et al. 2024	0.73 (0.42-0.88)	0.87 (0.78-0.93)

Supplementary Table 1.4. *Numerical estimates of vectorial capacity reduction for unwashed and washed/aged nets (posterior mean and 95% credible intervals), compared to untreated nets as control. Assenga et al.[3], Kibondo et al. [4], BIT055, BIT080[5], Odufuwa et al.[6], Martin et al.[7], N'Guessan et al.[8] and Sovegnon et al.[9] Holding time is 24 hours for BIT055 and Odufuwa et al.[6] and 72 hours for all other trials.*

2. Sample sizes and characteristics of all EHT data

Net type	Washed status	Total	UA	UD	FA	FD
**Assenga et al. 2025, Cote d'Ivoire**						
Control	Unwashed	804	403	43	350	8
	Washed 20x	933	534	45	352	2
Interceptor G2	Unwashed	580	108	379	25	68
	Washed 20x	1026	185	598	124	119
MagNet	Unwashed	726	380	175	118	53
	Washed 20x	926	571	209	126	20
Olyset Plus	Unwashed	604	340	209	25	30
	Washed 20x	1050	614	222	163	51
**Assenga et al. 2025, Tanzania**						
Control	.	1796	1505	41	245	5
Interceptor G2	Unwashed	967	476	443	41	7
	Washed 20x	1540	934	483	90	33
MagNet	Unwashed	1667	1005	626	30	6
	Washed 20x	2040	1363	614	59	4
Olyset Plus	Unwashed	780	485	277	14	4
	Washed 20x	1189	752	401	26	10
**BIT055**						
Control	.	1601	1177	70	335	19
Olyset	Unwashed	1510	868	577	47	18
	Washed 20x	1687	1081	506	78	22
Olyset Plus	Unwashed	1489	758	700	12	19
	Washed 20x	1929	1199	646	54	30
**BIT059**						
Control	.	1277	996	136	136	9
Olyset Plus	Unwashed	3154	1735	1376	24	19
	Washed 20x	4202	2795	1333	48	26
PermaNet2.0	Unwashed	2730	1853	760	107	10
	Washed 20x	4271	2926	1180	123	42
**BIT080**						
Control	.	4461	4130	29	301	1
Interceptor G2	Unwashed	1095	574	490	26	5
	Washed 20x	2786	1720	964	66	36
MiraNet	Unwashed	4501	3113	1254	114	20
	Washed 20x	4414	3107	1134	113	60

Supplementary Table 2.1. *Total numbers of mosquitoes for all experimental hut trials considered. Each quantity refers to total value summing over all nights in each experiment for each arm. UA: Unfed alive ; UD: Unfed dead ; FA: Fed alive ; FD: Fed dead.*

Net type	Washed status	Total	UA	UD	FA	FD
**Kibondo et al. 2022**						
Control	.	612	456	74	76	6
Interceptor	Unwashed	620	389	214	12	5
	Washed 20x	1934	1218	659	45	12
Interceptor G2	Unwashed	690	321	346	10	13
	Washed 20x	1599	769	776	28	26
**Martin et al. 2024, *An. funestus***						
Control	.	228	145	10	71	2
Interceptor	New	38	23	6	8	1
	36m	47	35	6	6	0
Interceptor G2	New	36	13	17	3	3
	36m	40	26	7	7	0
Olyset Plus	New	53	26	19	7	1
	36m	57	39	7	11	0
**Martin et al. 2024, *An. gambiae***						
Control	.	398	260	15	118	5
Interceptor	New	75	53	8	14	0
	36m	44	26	7	11	0
Interceptor G2	New	66	21	29	9	7
	36m	55	42	6	6	1
Olyset Plus	New	59	23	29	4	3
	36m	67	41	15	11	0
**Nguessan et al. 2016**						
Control	.	673	247	31	394	1
Interceptor	Unwashed	631	354	129	139	9
	Washed 20x	950	514	128	291	17
Interceptor G2	Unwashed	697	145	391	85	76
	Washed 20x	929	224	446	138	121
**Sovegnon et al. 2024, 1**						
Control	.	241	21	77	125	18
Interceptor	New	133	1	61	61	10
	24m	157	2	89	55	11
**Sovegnon et al. 2024, 2**						
Control	.	63	21	13	28	1
Interceptor G2	New	74	5	52	6	11
	24m	59	10	35	7	7

Supplementary Table 2.1 (cont.). *Total numbers of mosquitoes for all experimental hut trials considered. Each quantity refers to total value summing over all nights in each experiment for each arm. UA: Unfed alive ; UD: Unfed dead ; FA: Fed alive ; FD: Fed dead.*

Net type	Wash status	Fed	Dead	Total	UA	UD	FA	FD
**Assenga et al. 2025, Cote d'Ivoire**								
Control	Unwashed	0.44 (0-1)	0.05 (0-0.27)	7 (0-19)	4 (0-11)	0 (0-3)	3 (0-10)	0 (0-1)
	20x	0.37 (0-0.84)	0.04 (0-0.2)	9 (0-20)	5 (0-12)	0 (0-2)	3 (0-10)	0 (0-0)
Interceptor G2	Unwashed	0.16 (0-0.65)	0.72 (0.08-1)	5 (0-15)	1 (0-4)	4 (0-11)	0 (0-1)	1 (0-3)
	20x	0.23 (0-0.68)	0.68 (0.12-1)	10 (0-25)	2 (0-6)	6 (0-17)	1 (0-6)	1 (0-5)
MagNet	Unwashed	0.21 (0-0.7)	0.31 (0-0.85)	7 (0-18)	4 (0-11)	2 (0-6)	1 (0-5)	0 (0-3)
	20x	0.17 (0-0.6)	0.23 (0-0.67)	9 (0-23)	5 (0-15)	2 (0-7)	1 (0-5)	0 (0-1)
Olyset Plus	Unwashed	0.1 (0-0.5)	0.42 (0-1)	6 (0-15)	3 (0-10)	2 (0-6)	0 (0-1)	0 (0-2)
	20x	0.2 (0-0.62)	0.26 (0-0.69)	10 (0-24)	6 (0-16)	2 (0-7)	2 (0-6)	0 (0-2)
**Assenga et al. 2025, Tanzania**								
Control	.	0.18 (0-0.68)	0.04 (0-0.32)	8 (0-31)	7 (0-27)	0 (0-2)	1 (0-5)	0 (0-0)
Interceptor G2	Unwashed	0.05 (0-0.29)	0.5 (0-1)	9 (0-30)	4 (0-18)	4 (0-15)	0 (0-3)	0 (0-1)
	20x	0.07 (0-0.3)	0.38 (0-0.91)	14 (0-47)	9 (0-32)	4 (0-16)	1 (0-5)	0 (0-2)
MagNet	Unwashed	0.04 (0-0.28)	0.42 (0-1)	15 (0-52)	9 (0-35)	6 (0-22)	0 (0-2)	0 (0-1)
	20x	0.05 (0-0.33)	0.3 (0-0.83)	19 (0-60)	13 (0-43)	6 (0-23)	1 (0-3)	0 (0-1)
Olyset Plus	Unwashed	0.07 (0-0.45)	0.39 (0-1)	7 (0-35)	4 (0-25)	3 (0-12)	0 (0-1)	0 (0-0)
	20x	0.06 (0-0.4)	0.35 (0-0.96)	11 (0-36)	7 (0-25)	4 (0-15)	0 (0-1)	0 (0-1)
**BIT055**								
Control	.	0.23 (0-0.64)	0.05 (0-0.26)	11 (0-25)	8 (0-20)	0 (0-3)	2 (0-7)	0 (0-1)
Olyset	Unwashed	0.05 (0-0.2)	0.35 (0-0.86)	21 (0-46)	12 (0-30)	8 (0-25)	1 (0-3)	0 (0-2)
	20x	0.07 (0-0.24)	0.31 (0-0.73)	23 (0-56)	15 (0-38)	7 (0-24)	1 (0-4)	0 (0-1)
Olyset Plus	Unwashed	0.04 (0-0.24)	0.43 (0-0.98)	21 (0-56)	11 (0-27)	10 (0-33)	0 (0-1)	0 (0-2)
	20x	0.07 (0-0.36)	0.34 (0-0.78)	27 (0-72)	17 (0-49)	9 (0-27)	1 (0-3)	0 (0-2)
**BIT059**								
Control	.	0.13 (0-0.48)	0.11 (0-0.41)	13 (0-34)	10 (0-28)	1 (0-6)	1 (0-5)	0 (0-1)
Olyset Plus	Unwashed	0.02 (0-0.09)	0.44 (0.01-0.87)	32 (0-69)	18 (0-42)	14 (0-35)	0 (0-1)	0 (0-1)
	Washed 20x	0.02 (0-0.11)	0.35 (0-0.77)	43 (0-95)	29 (0-75)	14 (0-34)	0 (0-2)	0 (0-1)
PermaNet2.0	Unwashed	0.06 (0-0.27)	0.27 (0-0.66)	28 (0-66)	19 (0-49)	8 (0-22)	1 (0-4)	0 (0-1)
	Washed 20x	0.04 (0-0.14)	0.28 (0-0.6)	44 (0-100)	30 (0-70)	12 (0-35)	1 (0-5)	0 (0-3)
**BIT080**								
Control	.	0.07 (0-0.21)	0.01 (0-0.05)	46 (0-109)	42 (0-103)	0 (0-2)	3 (0-9)	0 (0-0)
Interceptor G2	Unwashed	0.04 (0-0.27)	0.45 (0-1)	11 (0-26)	6 (0-16)	5 (0-14)	0 (0-1)	0 (0-0)
	20x	0.04 (0-0.2)	0.36 (0-0.79)	28 (0-64)	18 (0-43)	10 (0-27)	1 (0-3)	0 (0-2)
MiraNet	Unwashed	0.03 (0-0.13)	0.29 (0-0.68)	46 (0-118)	32 (0-89)	13 (0-38)	1 (0-5)	0 (0-1)
	20x	0.04 (0-0.18)	0.27 (0-0.58)	45 (0-97)	32 (0-70)	12 (0-31)	1 (0-5)	1 (0-9)

Supplementary Table 2.2. *Summary statistics for all experimental hut trials. Each quantity refers to average value over all nights in the experiment, "Fed" and "Dead" correspond to respectively to (FA+FD)/Total and (UD+FD)/Total. Uncertainty intervals correspond to the mean value ± 2 standard deviations (capped to the [0-1] intervals for the proportions of fed and dead). UA: Unfed alive ; UD: Unfed dead ; FA: Fed alive ; FD: Fed dead.*

Net type	Wash status	Fed	Dead	Total	UA	UD	FA	FD
**Kibondo et al. 2022**								
Control	.	0.14 (0-0.48)	0.12 (0-0.54)	12 (0-33)	9 (0-26)	1 (0-7)	2 (0-6)	0 (0-1)
Interceptor	Unwashed	0.04 (0-0.26)	0.32 (0-0.86)	12 (0-33)	8 (0-21)	4 (0-17)	0 (0-2)	0 (0-1)
	20x	0.03 (0-0.12)	0.34 (0-0.81)	39 (0-83)	24 (0-60)	13 (0-36)	1 (0-4)	0 (0-2)
Interceptor G2	Unwashed	0.04 (0-0.2)	0.47 (0-0.95)	14 (0-33)	6 (0-15)	7 (0-20)	0 (0-1)	0 (0-2)
	20x	0.03 (0-0.12)	0.5 (0.04-0.97)	32 (1-63)	15 (0-36)	16 (0-38)	1 (0-3)	1 (0-2)
**Martin et al. 2024, *An. funestus***								
Control	.	0.35 (0-1)	0.05 (0-0.44)	1 (0-3)	0 (0-2)	0 (0-0)	0 (0-1)	0 (0-0)
Interceptor	New	0.24 (0-0.95)	0.24 (0-1)	1 (0-3)	0 (0-2)	0 (0-1)	0 (0-1)	0 (0-0)
	36m	0.16 (0-0.87)	0.12 (0-0.73)	0 (0-3)	0 (0-2)	0 (0-1)	0 (0-1)	0 (0-0)
Interceptor G2	New	0.14 (0-0.79)	0.66 (0-1)	0 (0-3)	0 (0-1)	0 (0-1)	0 (0-1)	0 (0-0)
	36m	0.13 (0-0.71)	0.24 (0-1)	0 (0-3)	0 (0-2)	0 (0-1)	0 (0-1)	0 (0-0)
Olyset Plus	New	0.15 (0-0.81)	0.38 (0-1)	1 (0-4)	0 (0-2)	0 (0-2)	0 (0-1)	0 (0-0)
	36m	0.18 (0-0.87)	0.14 (0-0.78)	0 (0-3)	0 (0-2)	0 (0-1)	0 (0-1)	0 (0-0)
**Martin et al. 2024, *An. gambiae***								
Control	.	0.34 (0-1)	0.06 (0-0.49)	1 (0-4)	1 (0-3)	0 (0-0)	0 (0-1)	0 (0-0)
Interceptor	New	0.18 (0-0.79)	0.11 (0-0.66)	1 (0-5)	1 (0-4)	0 (0-1)	0 (0-1)	0 (0-0)
	36m	0.27 (0-1)	0.12 (0-0.62)	0 (0-2)	0 (0-2)	0 (0-1)	0 (0-1)	0 (0-0)
Interceptor G2	New	0.3 (0-1)	0.51 (0-1)	1 (0-4)	0 (0-2)	0 (0-3)	0 (0-1)	0 (0-1)
	36m	0.08 (0-0.58)	0.12 (0-0.7)	0 (0-3)	0 (0-2)	0 (0-1)	0 (0-1)	0 (0-0)
Olyset Plus	New	0.11 (0-0.68)	0.49 (0-1)	1 (0-4)	0 (0-2)	0 (0-3)	0 (0-1)	0 (0-0)
	36m	0.2 (0-0.95)	0.2 (0-0.86)	1 (0-3)	0 (0-2)	0 (0-1)	0 (0-1)	0 (0-0)
**Nguessan et al. 2016**								
Control	.	0.63 (0.11-1)	0.04 (0-0.21)	9 (0-30)	3 (0-15)	0 (0-2)	5 (0-16)	0 (0-0)
Interceptor	Unwashed	0.28 (0-0.84)	0.23 (0-0.79)	9 (0-30)	5 (0-19)	2 (0-8)	2 (0-8)	0 (0-1)
	20x	0.39 (0-0.98)	0.19 (0-0.67)	13 (0-41)	7 (0-28)	2 (0-6)	4 (0-12)	0 (0-2)
Interceptor G2	Unwashed	0.24 (0-0.77)	0.75 (0.27-1)	10 (0-37)	2 (0-13)	6 (0-16)	1 (0-8)	1 (0-5)
	20x	0.32 (0-0.86)	0.64 (0.14-1)	13 (0-47)	3 (0-17)	6 (0-23)	2 (0-7)	2 (0-6)
**Sovegnon et al. 2024, 1**								
Control	.	0.54 (0-1)	0.44 (0-1)	4 (0-15)	0 (0-2)	1 (0-9)	2 (0-11)	0 (0-2)
Interceptor	New	0.55 (0-1)	0.49 (0-1)	3 (0-9)	0 (0-0)	1 (0-6)	1 (0-5)	0 (0-2)
	24m	0.45 (0-1)	0.61 (0-1)	3 (0-12)	0 (0-1)	2 (0-8)	1 (0-6)	0 (0-2)
**Sovegnon et al. 2024, 2**								
Control	.	0.47 (0-1)	0.26 (0-1)	1 (0-2)	0 (0-2)	0 (0-1)	0 (0-2)	0 (0-0)
Interceptor G2	New	0.23 (0-1)	0.82 (0.05-1)	1 (0-2)	0 (0-1)	1 (0-2)	0 (0-1)	0 (0-1)
	24m	0.2 (0-0.99)	0.75 (0-1)	1 (0-3)	0 (0-1)	0 (0-2)	0 (0-1)	0 (0-1)

Supplementary Table 2.2. (cont.). *Summary statistics for all experimental hut trials. Each quantity refers to average value over all nights in the experiment, "Fed" and "Dead" correspond to respectively to (FA+FD)/Total and (UD+FD)/Total. Uncertainty intervals correspond to the mean value ± 2 standard deviations (capped to the [0-1] intervals for the proportions of fed and dead). UA: Unfed alive ; UD: Unfed dead ; FA: Fed alive ; FD: Fed dead.*

3. Differences in vectorial capacity reduction between EHT arms

	EHT	Difference in vectorial capacity reduction (95% credible interval)		
		**Interceptor G2 - Pyrethroid**	**Olyset Plus - Pyrethroid**	**Interceptor G2 – Olyset Plus**
**1**	**Assenga et al.** [3] **(Tanzania)**	-0.8% – 2.5 %	-1.5% – 1.7 %	-1.15% – 2.71 %
**2**	**Kibondo et al.** [4]	1.6% – 7.8 %		
**3**	**BIT080**	3.3% – 8.5 %		
**4**	**BIT055**		0.7% – 3.0 %	
**5**	**Odufuwa et al.** [6]		8.1% – 15.3 %	
**6**	**Martin et al.**[7] **(*An. gambiae*)**	13.1% – 59.4 %	12.2% – 58.9 %	-4.3% – 5.0 %
**7**	**Martin et al.**[7] **(*An. funestus*)**	5.6% – 55.4 %	-2.9% – 50.7 %	-0.7% – 17.0 %
**8**	**Assenga et al.** [3] **(Côte d'Ivoire)**	7.6% – 12.6 %	2.5% – 7.7 %	4.0% – 6.1 %
**9**	**Nguessan et al.** [8]	10.6% – 16.6 %		

Supplementary Table 3. *Difference between vectorial capacity reduction estimates for 1000 parameter sets from the posterior distribution (2.5 and 97.5 quantiles of the distribution), to assess the difference in entomological efficacy between ITNs evaluated within the same EHT. Only unwashed/new ITNs are considered. Negative values are highlighted in red: these indicate that the interval contains the value 0 and therefore that we cannot conclude to a difference in entomological efficacy between the two ITNs given statistical uncertainty.*

4. ITN effectiveness cascades for each EHT independently

Supplementary Figure 4. *Cascades of ITN effectiveness, reflecting entomological efficacy from each experimental hut trial separately and setting-specific conditions of the Mosha et al.* [1,2] *randomized controlled trial. The various experimental huts considered are Assenga et al.*[3]*, Kibondo et al.* [4]*, BIT055, BIT080*[5]*, Odufuwa et al.* [6] *and Martin et al.* [7]*. Left panels (orange): Interceptor® G2. Right panels (blue): Olyset® Plus. Bars represent the mean and error bars represent the 95% credible intervals from EHT fitting of entomological efficacy, based on 1000 samples from each EHT and taking the ensemble over all EHT for each net type (see Methods section).*

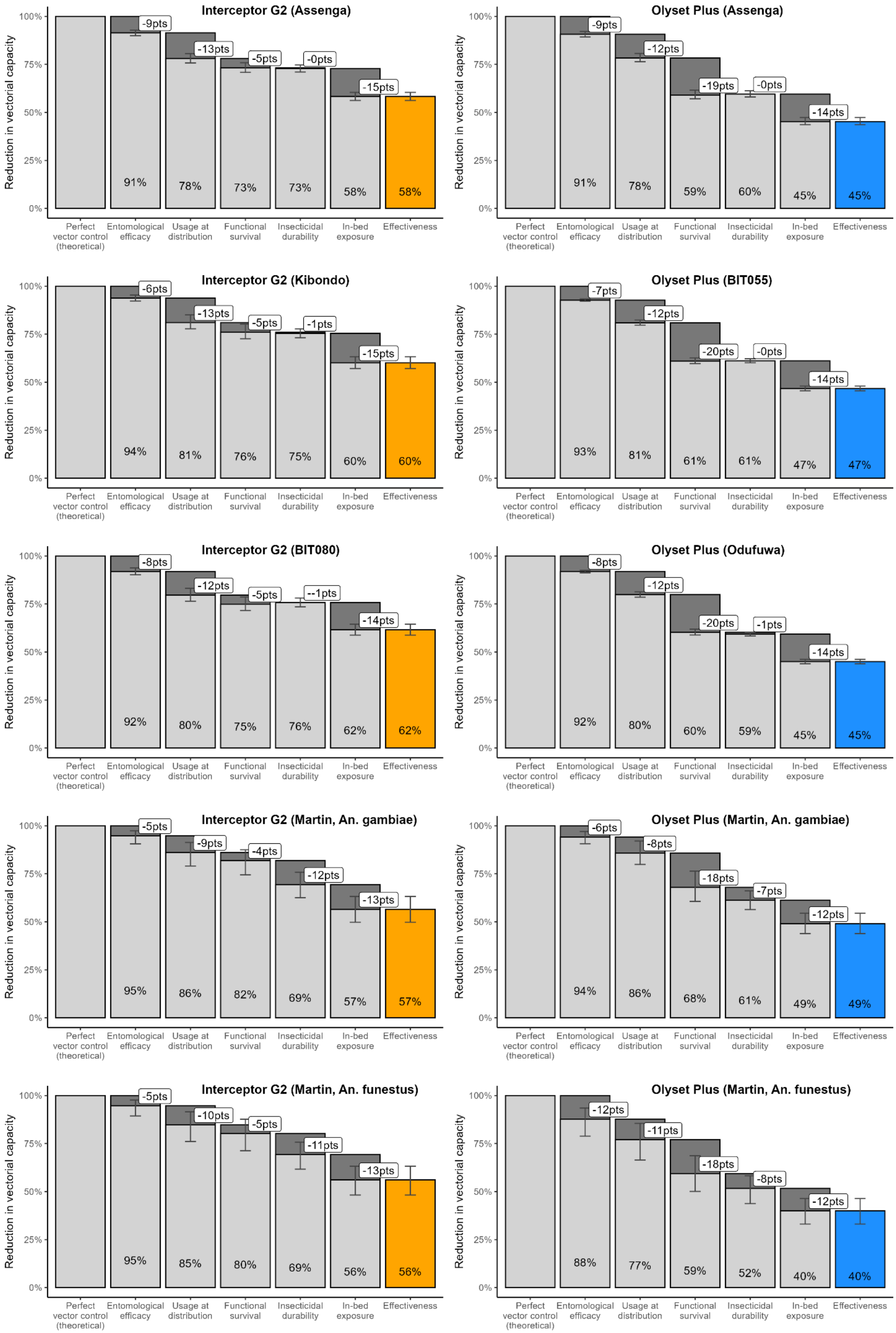

Interceptor G2 (Assenga)
Reduction in vectorial capacity
100%
75%
50%
25%
0%
-9pts
-13pts
-5pts
-0pts
-15pts
91%
78%
73%
73%
58%
58%
Perfect vector control (theoretical)
Entomological efficacy
Usage at distribution
Functional survival
Insecticidal durability
In-bed exposure
Effectiveness
Olyset Plus (Assenga)
Reduction in vectorial capacity
-9pts
-12pts
-19pts
-0pts
-14pts
91%
78%
59%
60%
45%
45%
Interceptor G2 (Kibondo)
Reduction in vectorial capacity
-6pts
-13pts
-5pts
-1pts
-15pts
94%
81%
76%
75%
60%
60%
Olyset Plus (BIT055)
Reduction in vectorial capacity
-7pts
-12pts
-20pts
-0pts
-14pts
93%
81%
61%
61%
47%
47%
Interceptor G2 (BIT080)
Reduction in vectorial capacity
-8pts
-12pts
-5pts
--1pts
-14pts
92%
80%
75%
76%
62%
62%
Olyset Plus (Odufuwa)
Reduction in vectorial capacity
-8pts
-12pts
-20pts
-1pts
-14pts
92%
80%
60%
59%
45%
45%
Interceptor G2 (Martin, An. gambiae)
Reduction in vectorial capacity
-5pts
-9pts
-4pts
-12pts
-13pts
95%
86%
82%
69%
57%
57%
Olyset Plus (Martin, An. gambiae)
Reduction in vectorial capacity
-6pts
-8pts
-18pts
-7pts
-12pts
94%
86%
68%
61%
49%
49%
Interceptor G2 (Martin, An. funestus)
Reduction in vectorial capacity
-5pts
-10pts
-5pts
-11pts
-13pts
95%
85%
80%
69%
56%
56%
Olyset Plus (Martin, An. funestus)
Reduction in vectorial capacity
-12pts
-11pts
-18pts
-8pts
-12pts
88%
77%
59%
52%
40%
40%

5. Sensitivity analysis on cascade order

	Point difference (min-max)	Rank (min-max)
**Interceptor G2**		
Insecticidal durability	3-5	3-4
In-bed exposure	12-15	1-2
Usage at distribution	12-15	1-2
Functional survival	4-5	3-4
**Olyset Plus**		
Insecticidal durability	2-3	4-4
In-bed exposure	14-17	1-2
Usage at distribution	11-13	3-3
Functional survival	15-20	1-2

Supplementary Table 5.1. *Sensitivity analysis on the ordering of the cascade, taking the average across the EHT conducted in Tanzania. Only the posterior maximum for EHT was used. Reading note: Across all permutations of the cascade items, the point difference associated with "Insecticide durability" was between 3 and 5 points for Interceptor G2. This factor was ranked as 3rd or 4th most important among the 4 factors considered.*

	Interceptor G2		Olyset Plus	
	Point difference (min-max)	Rank (min-max)	Point difference (min-max)	Rank (min-max)
**Assenga et al.**				
Insecticidal durability	0-1	4-4	-1--1	4-4
In-bed exposure	13-16	1-2	14-18	1-2
Usage at distribution	13-16	1-2	11-14	3-3
Functional survival	4-5	3-3	16-21	1-2
**Kibondo et al.**				
Insecticidal durability	0-1	4-4		
In-bed exposure	13-16	1-2		
Usage at distribution	13-16	1-2		
Functional survival	4-5	3-3		
**Odufuwa et al.**				
Insecticidal durability	-2-0	4-4		
In-bed exposure	12-15	1-2		
Usage at distribution	12-15	1-2		
Functional survival	4-5	3-3		
**BIT055**				
Insecticidal durability			0-1	4-4
In-bed exposure			14-18	1-2
Usage at distribution			11-14	3-3
Functional survival			16-21	1-2
**BIT059**				
Insecticidal durability			1-1	4-4
In-bed exposure			14-18	1-2
Usage at distribution			11-14	3-3
Functional survival			16-20	1-2
**Martin et al. (*An. funestus*)**				
Insecticidal durability	8-11	2-3	6-9	4-4
In-bed exposure	10-15	1-3	12-15	1-2
Usage at distribution	10-15	1-3	10-12	3-3
Functional survival	4-5	4-4	13-18	1-2
**Martin et al. (*An. gambiae*)**				
Insecticidal durability	8-13	1-3	4-6	4-4
In-bed exposure	9-14	1-3	13-16	1-2
Usage at distribution	9-14	1-3	9-13	3-3
Functional survival	3-5	4-4	15-19	1-2

Supplementary Table 5.2. *Sensitivity analysis on the ordering of the cascade, for each EHT conducted in Tanzania. Only the posterior maximum for EHT was used. Reading note: Across all permutations of the cascade items, the point difference associated with "Insecticide durability" was between 0 and 1 points for Interceptor G2 in the data from Assenga et al. 2025 (Tanzania). This factor was always ranked 4th most important among the 4 factors considered.*

6. Description of the unpublished experimental hut trials

Parameters	Description BIT055	Description BIT080
Time period	12/11/2020 to 22/12/2020	05/04/2023 to 29/05/2023
Mosquito resistance in tube test	0.75% Permethrin: 16% mortality at 24 hours<br>0.75% Permethrin after 4% PBO: 65% mortality at 24 hours	0.75% Permethrin: 13% mortality at 24 hours<br>0.05% Alpha-cypermethrin: 5% mortality at 24 hours<br>0.75% Permethrin after 4% PBO: 99% mortality at 24 hours<br>0.05% Alpha-cypermethrin after 4% PBO: 98% mortality at 24 hours
Location	8.385ºS and 36.670ºE Lupiro, Tanzania	
Design	Comparative efficacy study using a Latin square design	
Arm	UNWASHED<br>1. Olyset® Plus (permethrin and PBO)<br>2. Olyset® (permethrin)<br>3. Negative control (untreated net)<br>4. Investigational intervention arm 1<br>5. Investigational intervention arm 2<br>6. Investigational intervention arm 3<br>WASHED 20 times<br>7. Olyset® Plus (permethrin and PBO)<br>8. Olyset® (permethrin)<br>9. Negative control (untreated net)<br>10. Investigational intervention arm 1<br>11. Investigational intervention arm 2<br>12. Investigational intervention arm 3	UNWASHED<br>1. Interceptor® G2 (alpha-cypermethrin and CFP)<br>2. MiraNet® (alpha-cypermethrin)<br>3. Negative control (untreated net)<br>4. Investigational intervention arm 1<br>WASHED 20 times<br>5. Interceptor® G2 (alpha-cypermethrin and FP)<br>6. MiraNet® (alpha-cypermethrin)<br>7. Investigational intervention arm 1
Number of nights	N=72 per arm	N=98 per arm
Number of Huts	24	14
Species	Pyrethroid-resistant *Anopheles arabiensis*	
No. of mosquitoes	Median of 10 wild female mosquitoes/ hut-night	Median of 25 wild female mosquitoes/hut-night.
Exposure period	7 PM – 6 AM	
End points	1) Mosquito mortality at 24 hours<br>2) Proportion of mosquito blood-feeding	

Supplementary Table 6.1. *Trial specifications for BIT055 and BIT080*[5].

Parameters	Description BIT103
Time peiod	31/08/2023 to 27/12/2023
Mosquito resistance in tube test	0.05% Alphacypermethrin: 12% mortality at 24 hours<br>0.05% Alphacypermethrin after 4% PBO: 95% mortality at 24 hours
Location	8.385ºS and 36.670ºE Lupiro, Tanzania
Design	Comparative efficacy study using a Latin square design
Arm	UNWASHED<br>1. Interceptor G2 (chlorfenapyr and alphacypermethrin)<br>2. Olyset Plus (permethrin and PBO)<br>3. MAGNet (alphacypermethrin)<br>4. Negative control (untreated net)<br>5. Investigational intervention arm 1<br>6. Investigational intervention arm 2<br>7. Investigational intervention arm 3<br>8. Investigational intervention arm 4<br>9. Investigational intervention arm 5<br><br>WASHED 20 times<br>10. Interceptor G2 (chlorfenapyr and alphacypermethrin)<br>11. Olyset Plus (permethrin and PBO)<br>12. MAGNet (alphacypermethrin)<br>13. Negative control (untreated net)<br>14. Investigational intervention arm 1<br>15. Investigational intervention arm 2<br>16. Investigational intervention arm 3<br>17. Investigational intervention arm 4<br>18. Investigational intervention arm 5
Number of nights	N=108 per arm
Number of Huts	18
Species	Pyrethroid-resistant *Anopheles arabiensis*
No. of mosquitoes	Median 4 wild mosquitoes/ hut-night
Exposure period	7 PM – 6 AM
End points	1) Mosquito mortality at 24 hours<br>2) Mosquito mortality at 72 hours<br>3) Proportion of mosquito blood-feeding

Supplementary Table 6.2. *Trial specifications for Assenga et al.[3], Tanzania.*

7. Estimates of entomological indicators using 24h holding time

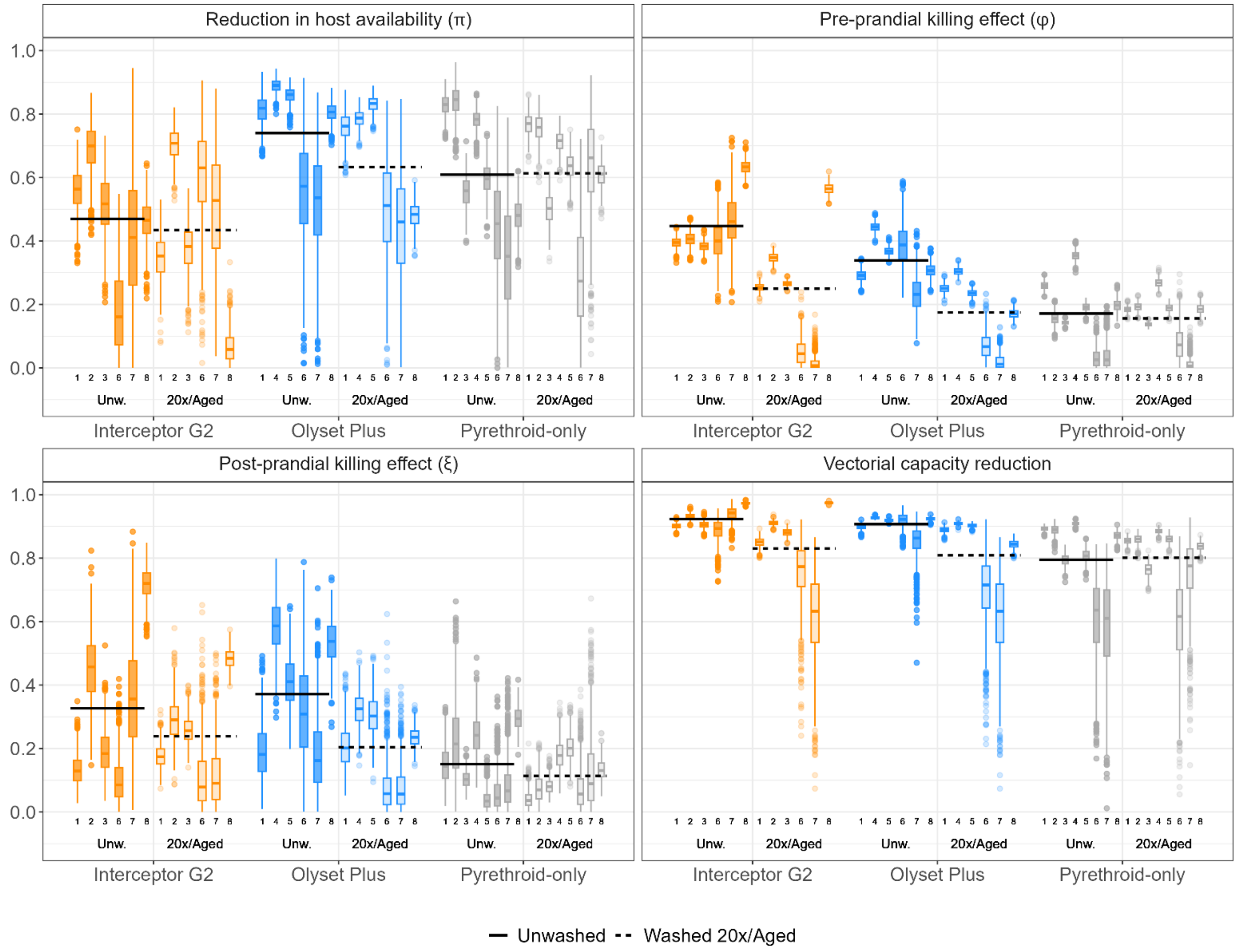

Supplementary Figure 7. *Estimates of entomological efficacy for unwashed and washed/aged nets compared to untreated nets as control, using 24h holding time for all studies. The black horizontal lines represent the average for unwashed nets (solid line) and 20-times washed/aged nets (dashed lines) across all experimental hut trials (EHT) per net type. Each boxplot corresponds to a specific EHT: 1) Assenga et al.[3] (Tanzania), 2) Kibondo et al. [4] (Tanzania), 3) BIT080[5] (Tanzania), 4) BIT055 (Tanzania), 5) Odufuwa et al. [6] (Tanzania), 6) Martin et al. [7] (An. gambiae, Tanzania), 7) Martin et al. [7] (An. funestus, Tanzania), 8) Assenga et al. [3] (Côte d'Ivoire). The data are derived from 1000 samples from the posterior distribution of each parameter. In each boxplot, the central line represents the median, the lower and upper hinges represent the 25th and 75th percentiles, and the whiskers extend to the largest and smallest values within 1.5 times the interquartile range from the hinges. Data points beyond the whiskers are plotted individually. "Unw." refers to unwashed nets (depicted darker boxplot shading) and "20x/Aged" refers to nets washed 20-times/aged (depicted with lighter boxplot shading).*

8. MCMC convergence diagnostics

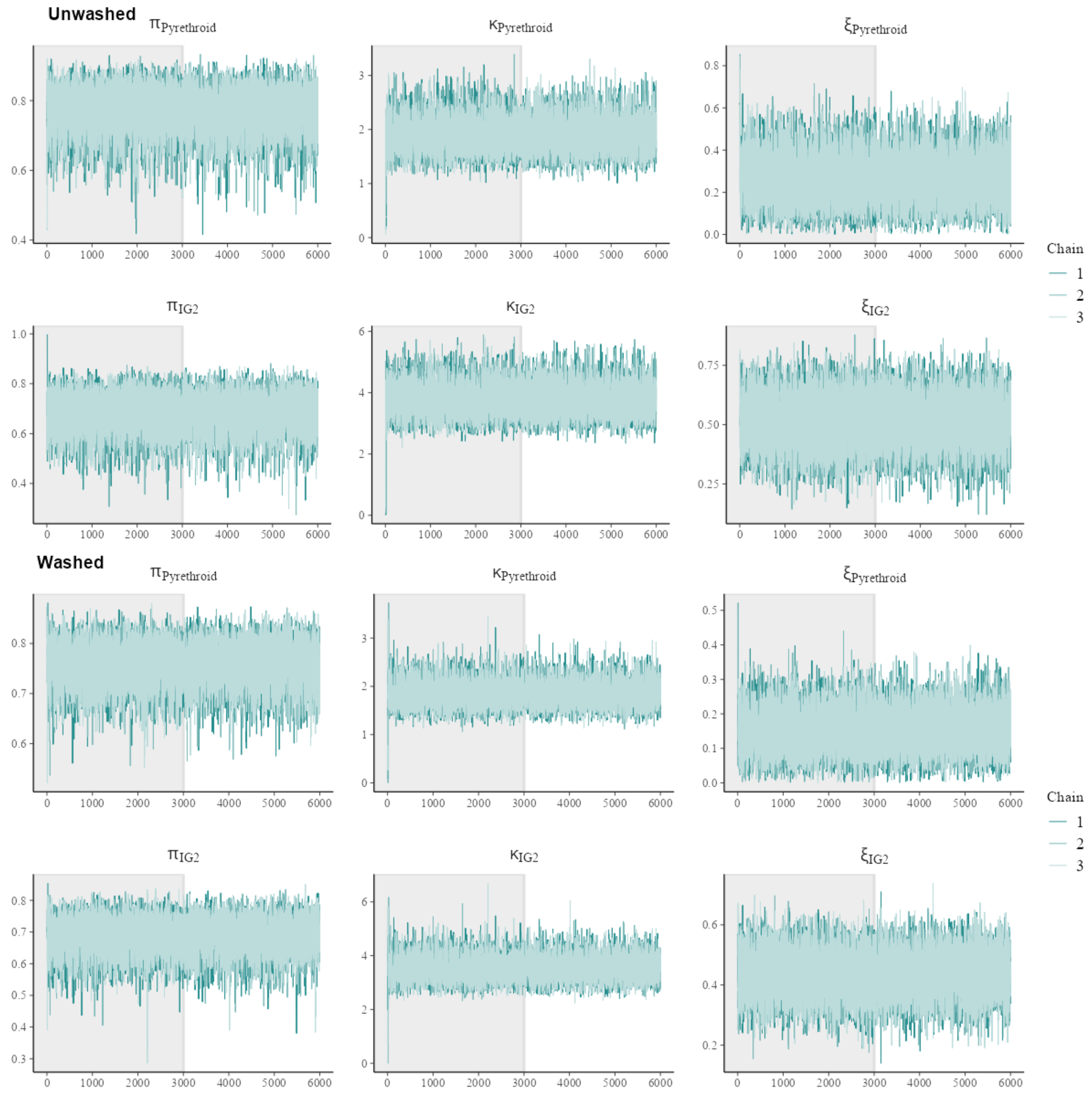

Supplementary Figure 8.1. Traceplots of the MCMC chains for the EHT trial by Kibondo et al. [4].

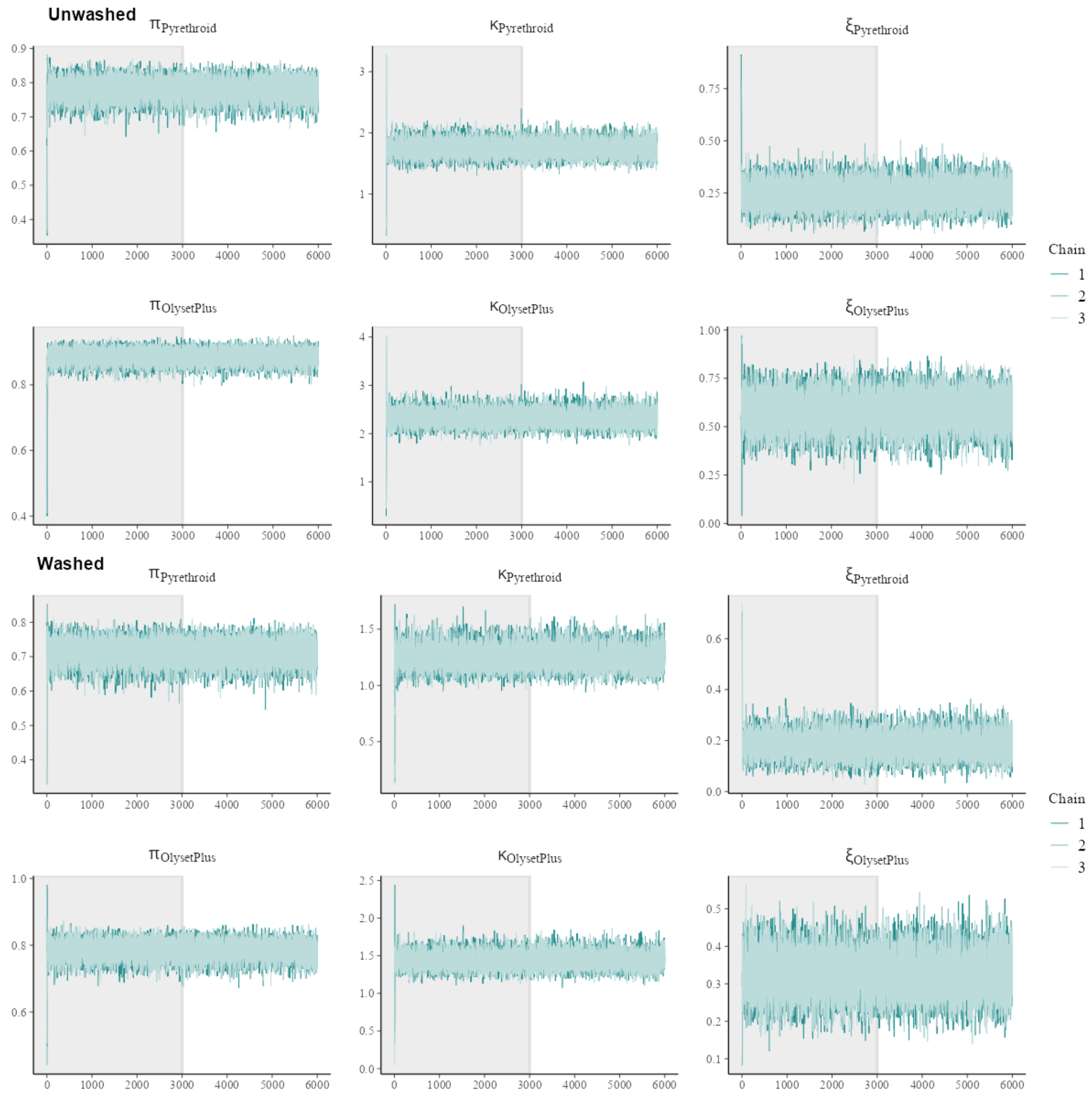

Supplementary Figure 8.2. Traceplots of the MCMC chains for the EHT trial BIT055.

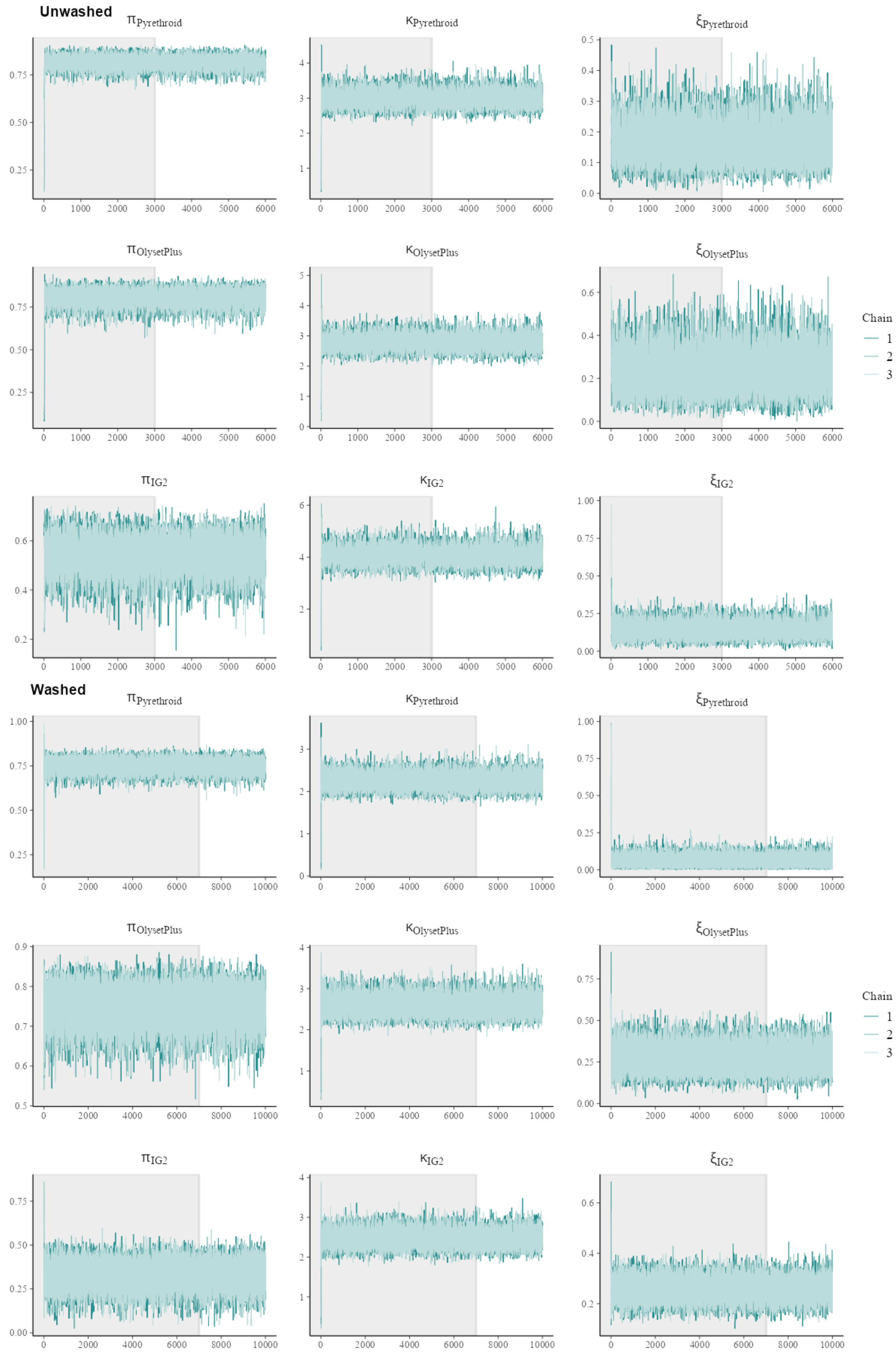

Supplementary Figure 8.3. Traceplots of the MCMC chains for the EHT trial by Assenga et al.[3] (Tanzania data).

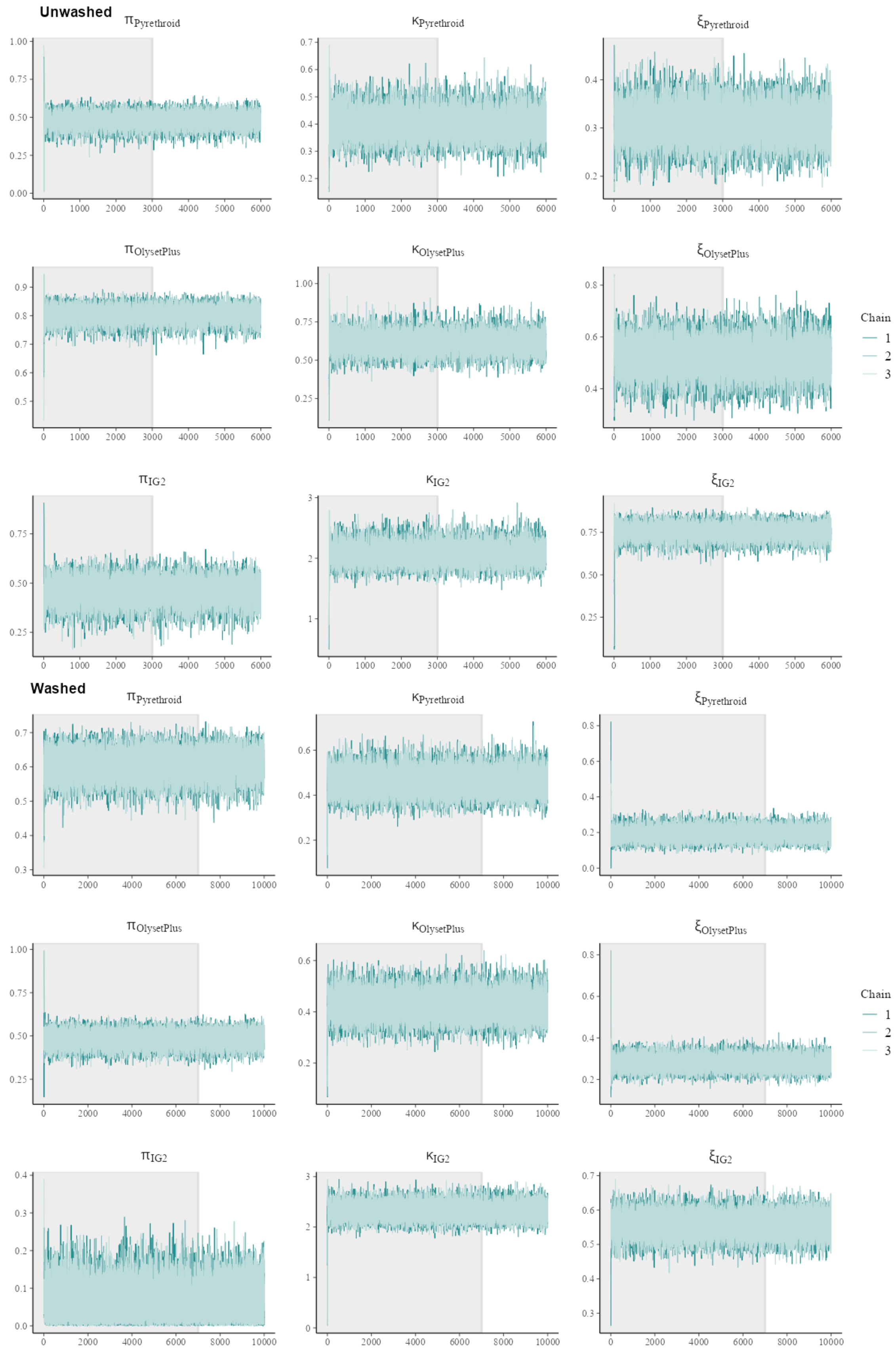

Supplementary Figure 8.4. Traceplots of the MCMC chains for the EHT trial by Assenga et al.[3] (Côte d'Ivoire data).

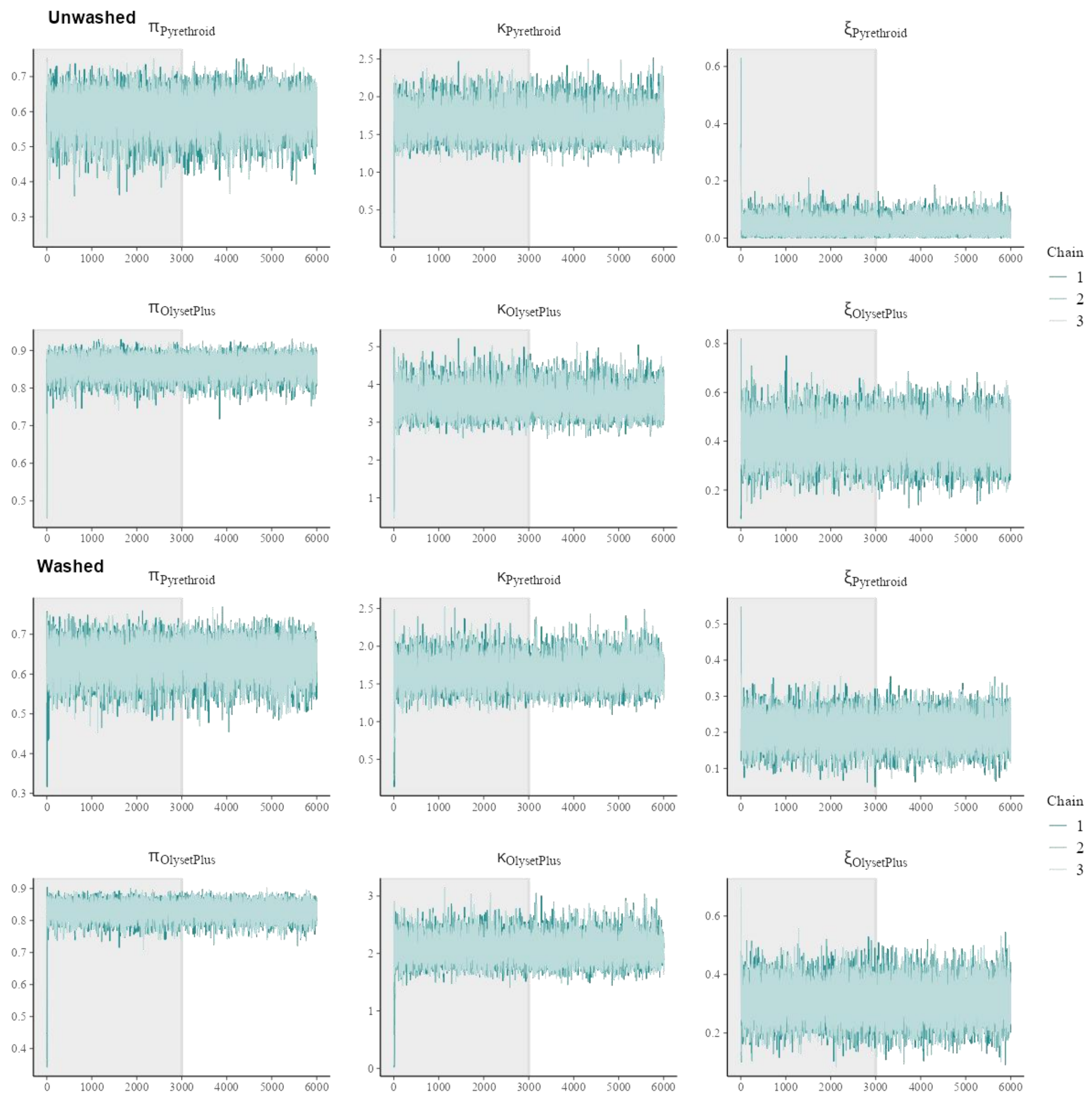

Supplementary Figure 8.5. Traceplots of the MCMC chains for the EHT trial by Odufuwa et al.[6].

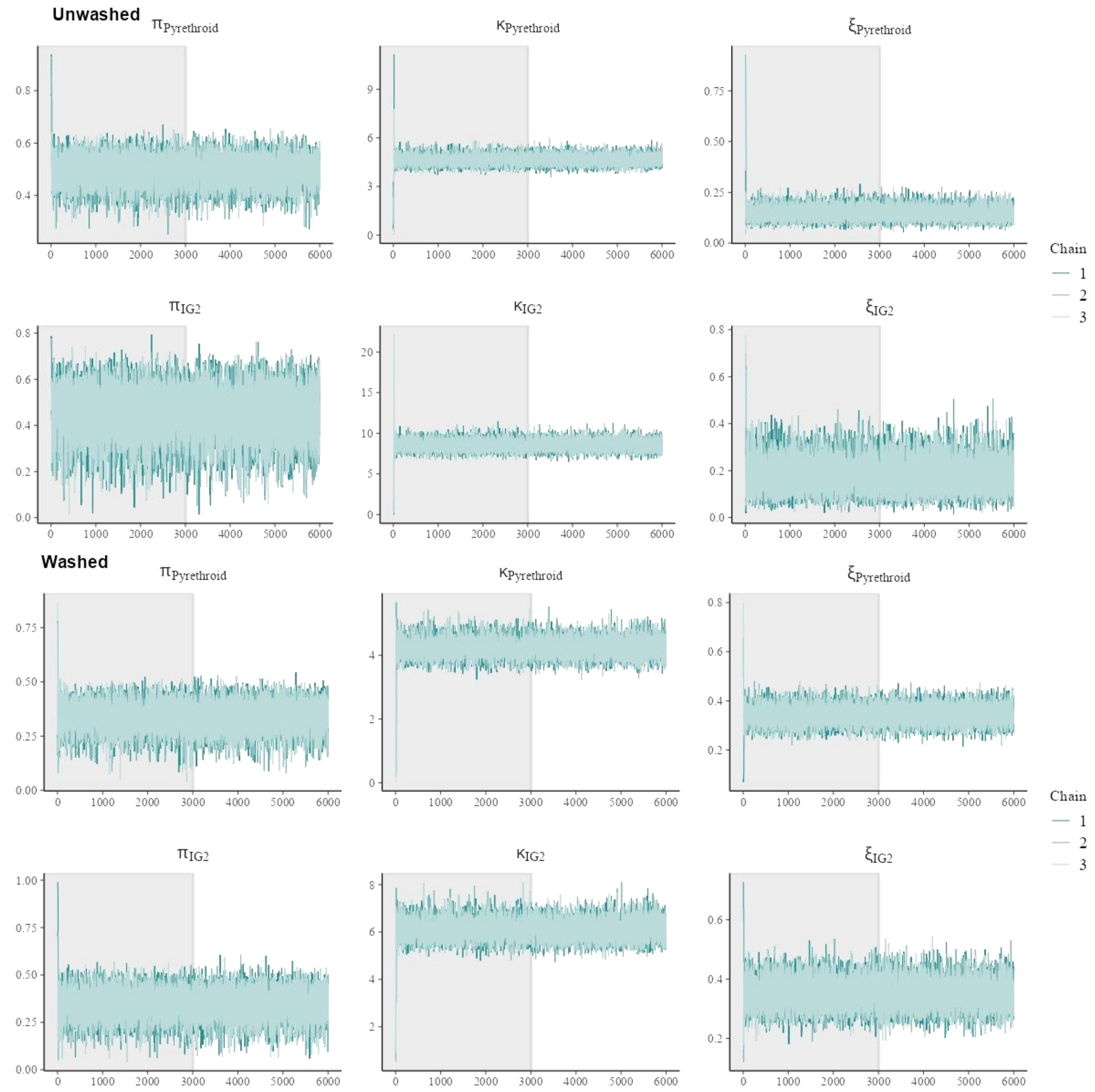

Supplementary Figure 8.6. Traceplots of the MCMC chains for EHT trial BIT080.

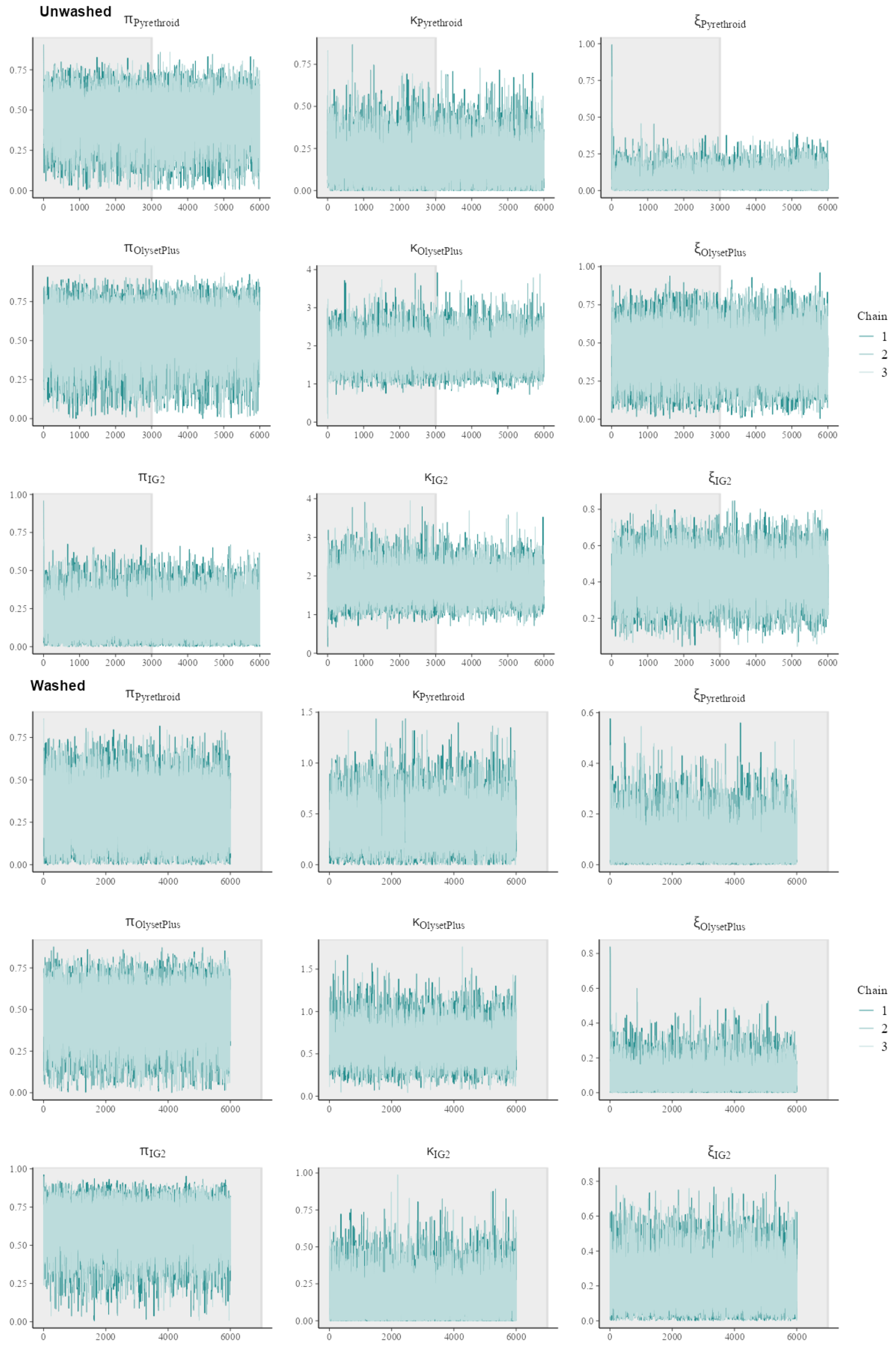

Supplementary Figure 8.7. Traceplots of the MCMC chains for the EHT trial by Martin et al.[7] (Anopheles gambiae)*.*

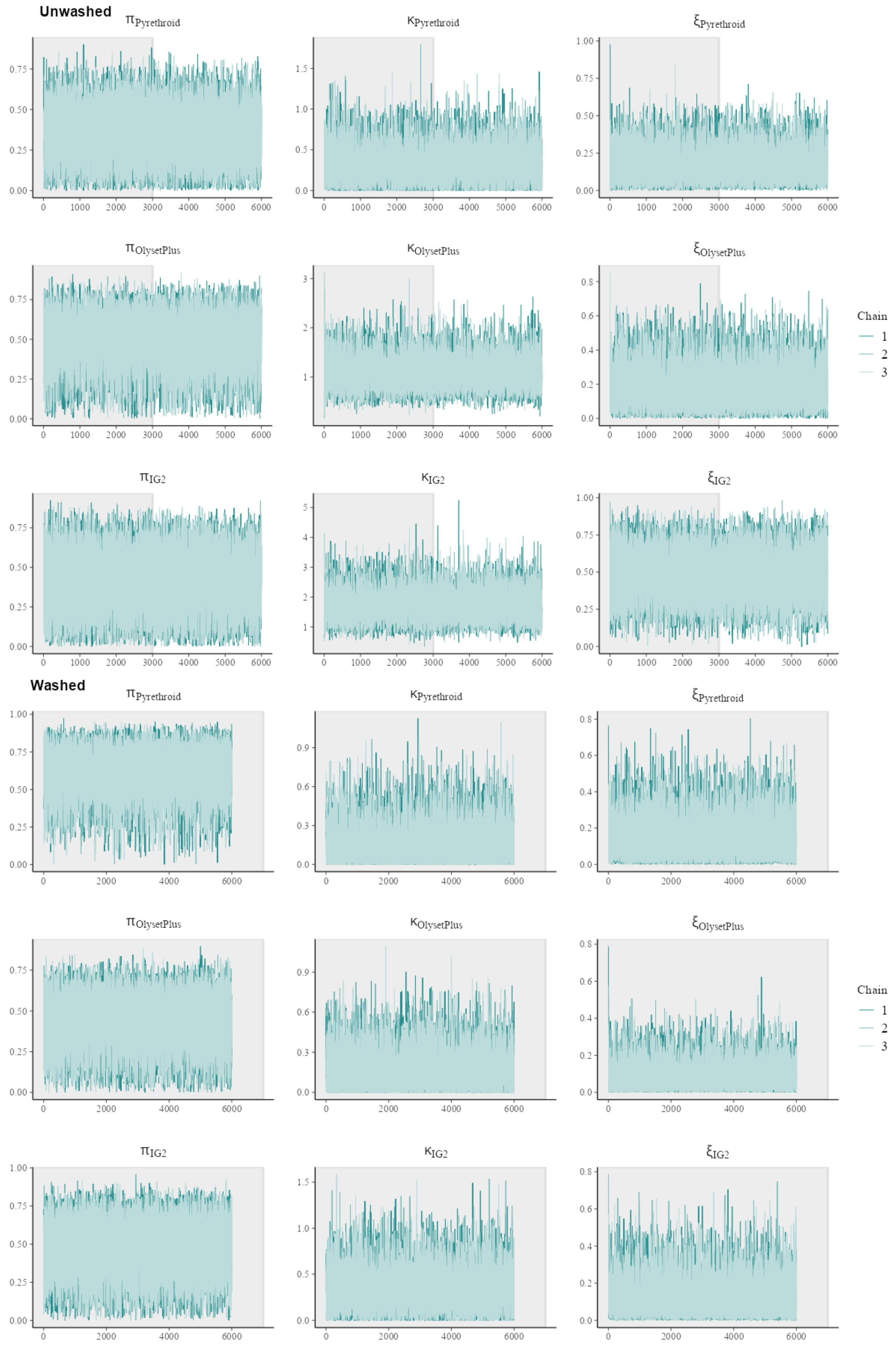

Supplementary Figure 8.8. Traceplots of the MCMC chains for the EHT trial by Martin et al.[7] *(*Anopheles funestus*).*

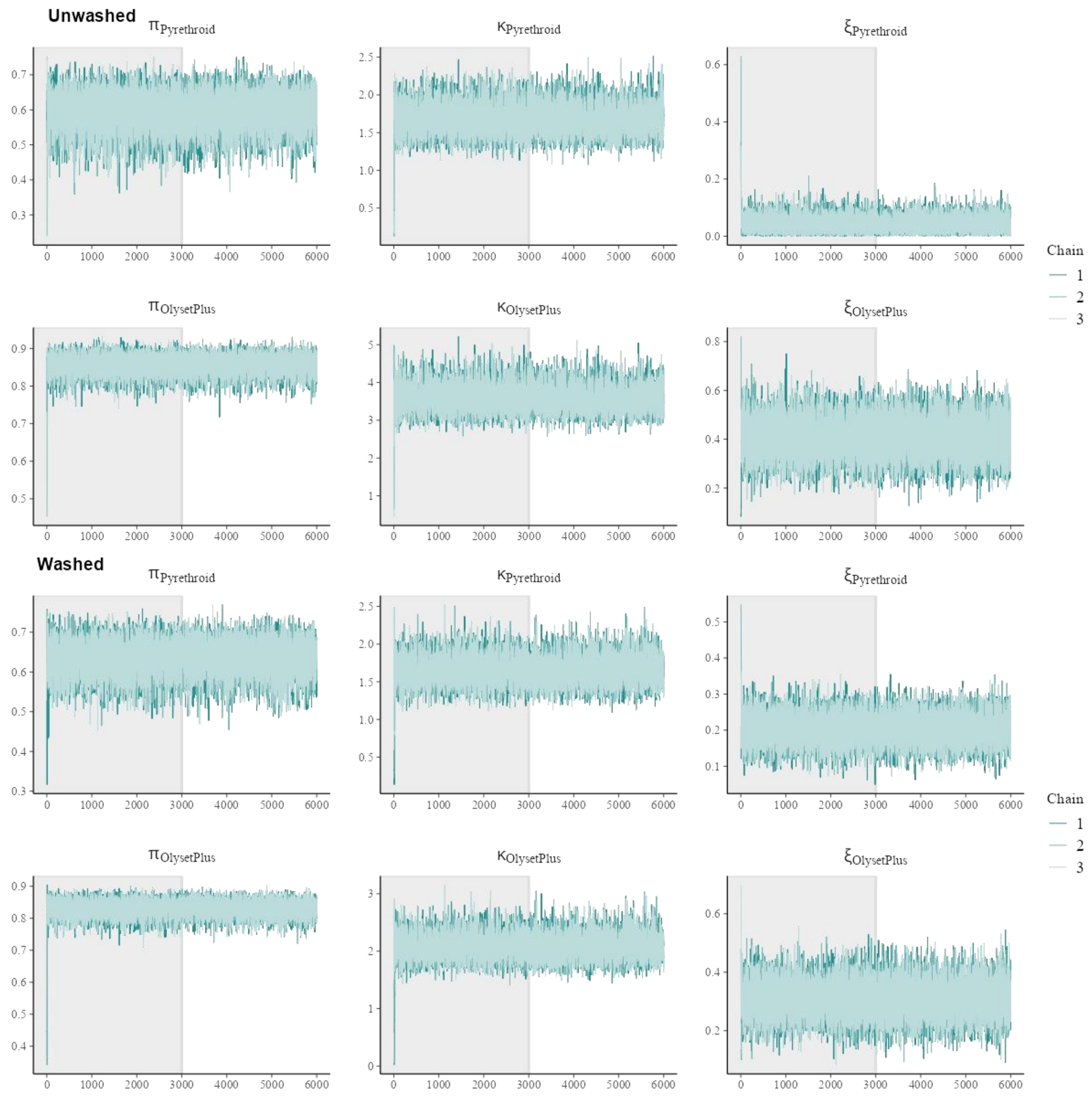

Supplementary Figure 8.9. Traceplots of the MCMC chains for the EHT trial by N'Guessan et al.[8].

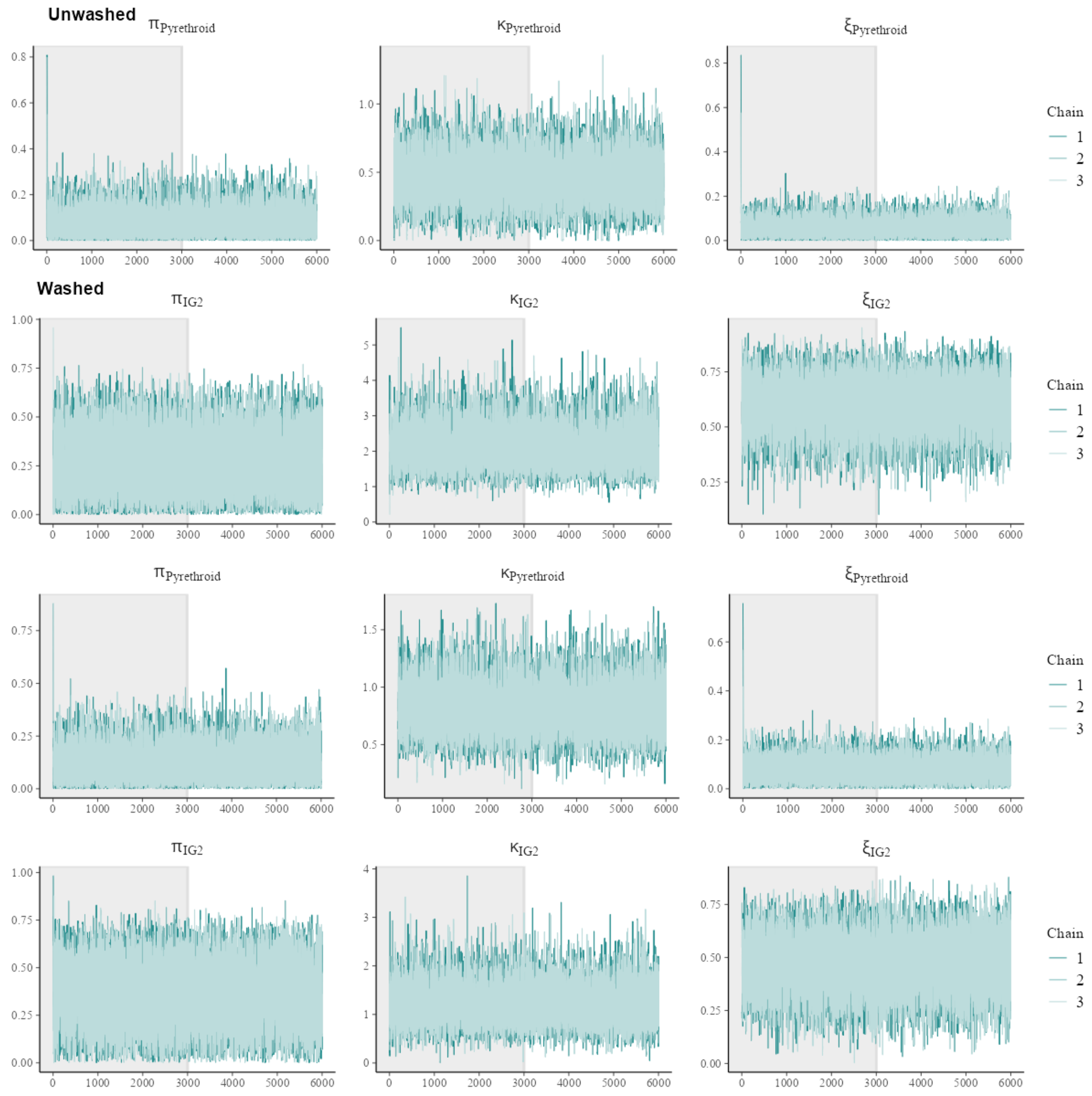

Supplementary Figure 8.10. Traceplots of the MCMC chains for EHT trial by Sovegnon et al.[9]*.*

9. Calibration methodology for RCT simulation.

The objective is to match the OpenMalaria simulations with data or estimates on *P. falciparum* prevalence. The methodology presented below is similar to the one in Lemant et al. [22].

Due to the limited number of data points and their large uncertainty, only one parameter is calibrated, namely the intensity of transmission for each geographical area (represented in OpenMalaria by the Entomological Inoculation Rate, EIR). Because of the properties of this parameter (finite range between 0 and 300 and little sensitivity of model outcomes to EIR differences below 1), a grid approach is used.

A grid of simulations sharing the same history of interventions but with a range of varying EIR values between 1 and 200.

We note X^{obs} the pre-intervention prevalence measured in the RCT with associated standard deviation $\sigma = \frac{\sqrt{X^{obs}(1-X^{obs})}}{n^2}$ where n is the reported sample size. For a given set of parameters θ (including the initial EIR), the associated OpenMalaria simulation for pre-intervention prevalence is noted $X^{sim,\theta}$. For each simulation in the grid, a pseudo-likelihood is computed to compare the predicted prevalence to the reported prevalence:

$$L\left(X^{obs}, X^{sim,\theta}\right) = \Phi_{X^{sim,\theta},\sigma_i}(X^{obs})$$

Where $\Phi_{\mu,\sigma}$ represents the probability density function of a normal distribution with mean μ and standard deviation σ. The EIR value with highest log-likelihood (EIR^{MLE}) is selected as point estimate.

An uncertainty estimate is computed using the methodology by [23], adapted to this particular use case. In [23], a modified profile likelihood approach is used, where a cut-off on the profile likelihood defines a confidence interval on the parameter of interest. The cut-off, based on the quantile of a Chi square distribution, is adjusted to account for uncertainty in the pseudo-likelihood function. For this, a quadratic approximation is fitted on the smoothed likelihood and the parameters of that quadratic function are used to calculate the adjustment factor.

This approach is extrapolated to our context and definition of the pseudo-likelihood. The method is applied using 10 stochastic replications with a loess smoother applied to the EIR values for which $\frac{|EIR^{MLE}-EIR|}{EIR^{MLE}} < 0.5$.

The EIR point estimate as well as the upper and lower uncertainty bounds are all simulated for the future scenarios, in order to propagate the uncertainty in transmission intensity due to prevalence data fitting.

10. Estimating ITN attrition and intervention usage from RCT surveys on ITN usage

In order to estimate the effective half-life of the ITNs functional survival, a Weibull decay function is fitted to the observed usage data, minimizing a least square distance between observed data and the following function:

$$\Delta_{c_0,L,\kappa}(t) = c_0 \exp\left(-\left(\frac{t}{L}\right)^{\kappa} \log(2)\right)$$

where t represents time and the parameters c_0 (initial ITN usage, between 0 and 1), L (effective half life, between 0 and 3) and κ (shape parameter, above 0) are estimated.

For the trial by Mosha et al. [1,2], the usage values for all household members of all ages were used between 3 months and 24 months post interventions, and the values for selected children for 30 and 36 months post-intervention (in the absence of data for all age groups in [2], and by digitizing Figure S2 using [10]). Due to the similarity between the estimates for children and all age groups, this assumption should not impact the obtained results.

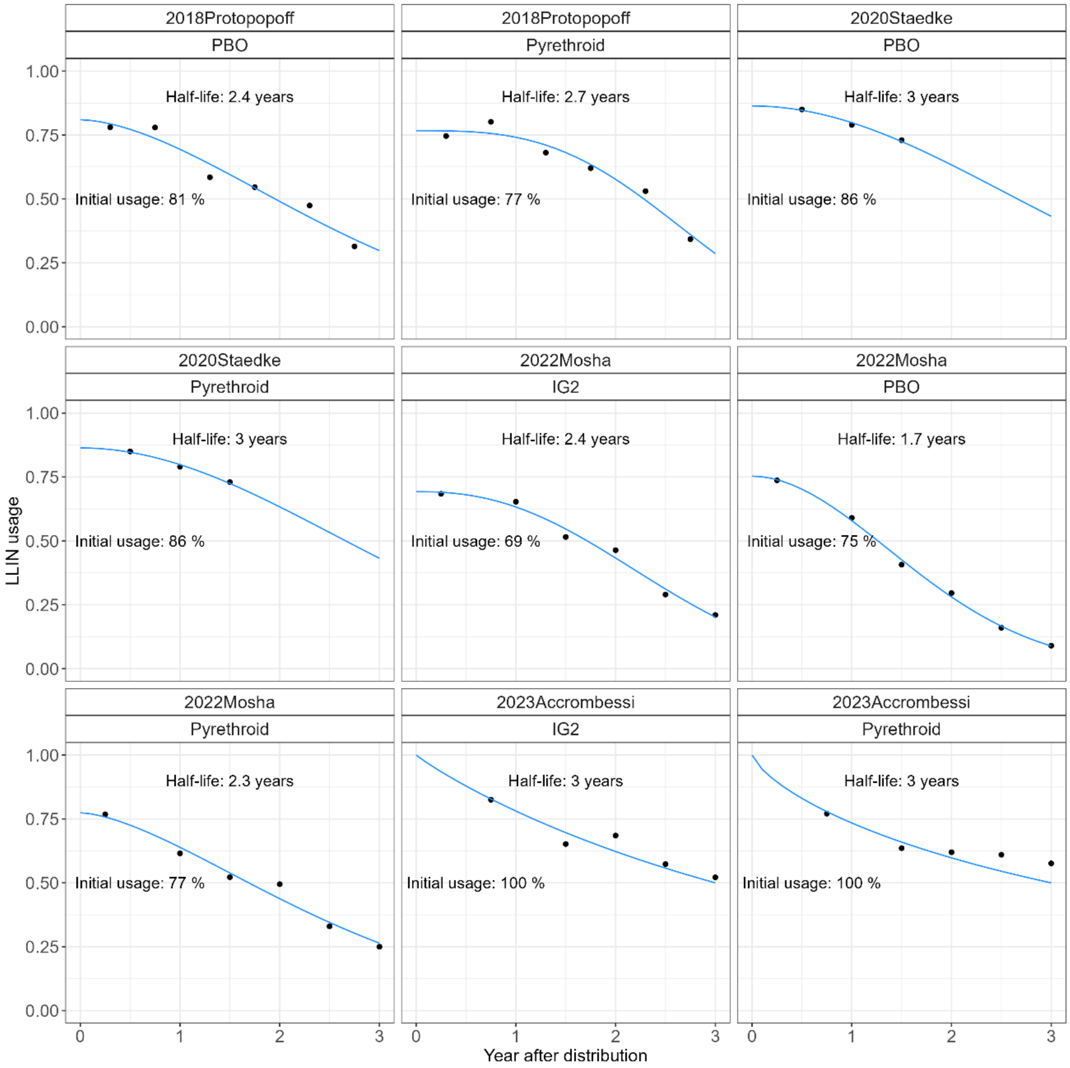

Supplementary Figure 10. *Reported ITN usage data in each RCT (dots) and fitted decay curve (blue curve). The RCTs considered are Mosha et al.[1,2], Protopopoff et al.[11,12], Accrombessi et al.[13,14] and Staedke et al.[15]. IG2: Interceptor G2. PBO: PBO-pyrehtroid ITNs (including Olyset Plus). Pyrehtroid: Pyrethroid-only ITNs.*

11. Activity patterns

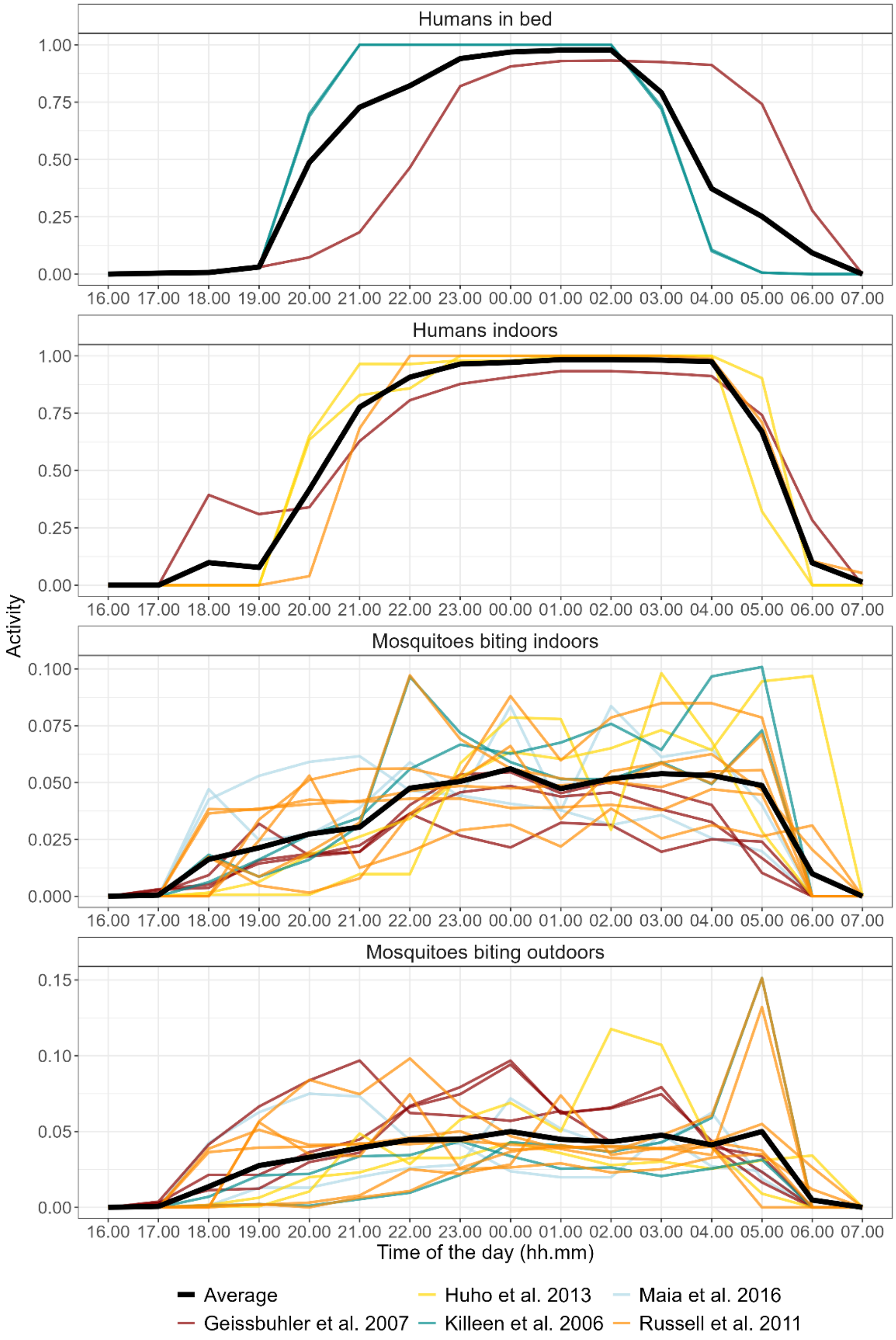

Supplementary Figure 11. *Input data used to calculate exposure coefficients, using the method by Golumbeanu et al. [16]. Input data refer to the studies [17–21] for mosquito rhythms and the studies [18–21] for human rhythms collected in Tanzania.*

References

1. Mosha, J. F. *et al.* Effectiveness and cost-effectiveness against malaria of three types of dual-active-ingredient long-lasting insecticidal nets (LLINs) compared with pyrethroid-only LLINs in Tanzania: a four-arm, cluster-randomised trial. *The Lancet* **399**, 1227–1241 (2022).
2. Mosha, J. F. *et al.* Effectiveness of long-lasting insecticidal nets with pyriproxyfen-pyrethroid, chlorfenapyr-pyrethroid, or piperonyl butoxide-pyrethroid versus pyrethroid only against malaria in Tanzania: final-year results of a four-arm, single-blind, cluster-randomised trial. *Lancet Infect. Dis.* **24**, 87–97 (2024).
3. Assenga, A. A. *et al.* PRONet Duo insecticide-treated net incorporated with chlorfenapyr and bifenthrin is superior to Interceptor® G2 nets against pyrethroid-resistant Anopheles gambiae sensu lato: a randomized experimental hut trial in Côte d'Ivoire and Tanzania using non-inferiority design. *Front. Malar.* **3**, (2025).
4. Kibondo, U. A. *et al.* Influence of testing modality on bioefficacy for the evaluation of Interceptor® G2 mosquito nets to combat malaria mosquitoes in Tanzania. *Parasit. Vectors* **15**, 124 (2022).
5. Odufuwa, O. G. & Moore, S. J. Yorkool® G5 – chlorfenapyr insecticide-treated nets demonstrated comparative non-inferiority efficacy to the first-in-class Interceptor® G2 in the experimental hut in Tanzania against wild Anopheles arabiensis mosquitoes. Preprint at (in prep).
6. Odufuwa, O. G. *et al.* The non-inferiority of piperonyl-butoxide Yorkool® G3 insecticide-treated nets compared to Olyset®Plus measured by Anopheles arabiensis mortality in experimental huts in Tanzania. *Malar. J.* **23**, 309 (2024).
7. Martin, J. L. *et al.* Bio-efficacy of field aged novel class of long-lasting insecticidal nets, against pyrethroid-resistant malaria vectors in Tanzania: A series of experimental hut trials. *PLOS Glob. Public Health* **4**, e0002586 (2024).

8. N'Guessan, R., Odjo, A., Ngufor, C., Malone, D. & Rowland, M. A Chlorfenapyr Mixture Net Interceptor® G2 Shows High Efficacy and Wash Durability against Resistant Mosquitoes in West Africa. *PLOS ONE* **11**, e0165925 (2016).
9. Sovegnon, P. M. *et al.* Efficacy of Interceptor G2, Royal Guard and PermaNet 3.0 against pyrethroid-resistant Anopheles gambiae s.l. from Za-Kpota, southern Benin: an experimental hut trial. *Parasit. Vectors* **17**, 300 (2024).
10. PlotDigitizer: Extract Data from Graph Image Online. *PlotDigitizer* https://plotdigitizer.com/.
11. Protopopoff, N. *et al.* Effectiveness of a long-lasting piperonyl butoxide-treated insecticidal net and indoor residual spray interventions, separately and together, against malaria transmitted by pyrethroid-resistant mosquitoes: a cluster, randomised controlled, two-by-two factorial design trial. *The Lancet* **391**, 1577–1588 (2018).
12. Protopopoff, N. *et al.* Effectiveness of piperonyl butoxide and pyrethroid-treated long-lasting insecticidal nets (LLINs) versus pyrethroid-only LLINs with and without indoor residual spray against malaria infection: third year results of a cluster, randomised controlled, two-by-two factorial design trial in Tanzania. *Malar. J.* **22**, 294 (2023).
13. Accrombessi, M. *et al.* Efficacy of pyriproxyfen-pyrethroid long-lasting insecticidal nets (LLINs) and chlorfenapyr-pyrethroid LLINs compared with pyrethroid-only LLINs for malaria control in Benin: a cluster-randomised, superiority trial. *The Lancet* (2023) doi:10.1016/S0140-6736(22)02319-4.
14. Accrombessi, M. *et al.* Effectiveness of pyriproxyfen-pyrethroid and chlorfenapyr-pyrethroid long-lasting insecticidal nets (LLINs) compared with pyrethroid-only LLINs for malaria control in the third year post-distribution: a secondary analysis of a cluster-randomised controlled trial in Benin. *Lancet Infect. Dis.* **0**, (2024).
15. Staedke, S. G. *et al.* Effect of long-lasting insecticidal nets with and without piperonyl butoxide on malaria indicators in Uganda (LLINEUP): a pragmatic, cluster-randomised trial embedded in a national LLIN distribution campaign. *The Lancet* **395**, 1292–1303 (2020).

16. Golumbeanu, M. *et al.* AnophelesModel: An R package to interface mosquito bionomics, human exposure and intervention effects with models of malaria intervention impact. 2023.10.17.562838 Preprint at https://doi.org/10.1101/2023.10.17.562838 (2023).

17. Maia, M. F. *et al.* A crossover study to evaluate the diversion of malaria vectors in a community with incomplete coverage of spatial repellents in the Kilombero Valley, Tanzania. *Parasit. Vectors* **9**, 451 (2016).

18. Geissbühler, Y. *et al.* Interdependence of domestic malaria prevention measures and mosquito-human interactions in urban Dar es Salaam, Tanzania. *Malar. J.* **6**, 126 (2007).

19. Killeen, G. F., Ross, A. & Smith, T. INFECTIOUSNESS OF MALARIA-ENDEMIC HUMAN POPULATIONS TO VECTORS. *Am. J. Trop. Med. Hyg.* **75**, 38–45 (2006).

20. Huho, B. *et al.* Consistently high estimates for the proportion of human exposure to malaria vector populations occurring indoors in rural Africa. *Int. J. Epidemiol.* **42**, 235–247 (2013).

21. Russell, T. L. *et al.* Increased proportions of outdoor feeding among residual malaria vector populations following increased use of insecticide-treated nets in rural Tanzania. *Malar. J.* **10**, 80 (2011).

22. Lemant, J. *et al.* Supporting evidence-based decisions about the geographic and demographic extensions of seasonal malaria chemoprevention in Benin: A modelling study. *PLOS Glob. Public Health* **5**, e0004509 (2025).

23. Ionides, E. L., Breto, C., Park, J., Smith, R. A. & King, A. A. Monte Carlo profile confidence intervals for dynamic systems. *J. R. Soc. Interface* **14**, 20170126 (2017).
