# Supplementary material for: Cascades of effectiveness of next-generation insecticide-treated nets against malaria, from entomological trials to real-life conditions
Source: Nat Commun. 2025 Dec 16;16:11162. doi: 10.1038/s41467-025-66130-y (PMC12708653; doi:10.1038/s41467-025-66130-y)
Supplement: Supplementary file 2 — Reporting Summary [file 41467_2025_66130_MOESM2_ESM.pdf]

nature portfolio

Corresponding author(s): Clara Champagne
Last updated by author(s): Oct 20, 2025

Reporting Summary

Nature Portfolio wishes to improve the reproducibility of the work that we publish. This form provides structure for consistency and transparency in reporting. For further information on Nature Portfolio policies, see our Editorial Policies and the Editorial Policy Checklist.

Statistics

For all statistical analyses, confirm that the following items are present in the figure legend, table legend, main text, or Methods section.

n/a	Confirmed	
☐	☒	The exact sample size (*n*) for each experimental group/condition, given as a discrete number and unit of measurement
☐	☒	A statement on whether measurements were taken from distinct samples or whether the same sample was measured repeatedly
☒	☐	The statistical test(s) used AND whether they are one- or two-sided<br>*Only common tests should be described solely by name; describe more complex techniques in the Methods section.*
☒	☐	A description of all covariates tested
☒	☐	A description of any assumptions or corrections, such as tests of normality and adjustment for multiple comparisons
☐	☒	A full description of the statistical parameters including central tendency (e.g. means) or other basic estimates (e.g. regression coefficient) AND variation (e.g. standard deviation) or associated estimates of uncertainty (e.g. confidence intervals)
☒	☐	For null hypothesis testing, the test statistic (e.g. *F*, *t*, *r*) with confidence intervals, effect sizes, degrees of freedom and *P* value noted<br>*Give P values as exact values whenever suitable.*
☐	☒	For Bayesian analysis, information on the choice of priors and Markov chain Monte Carlo settings
☒	☐	For hierarchical and complex designs, identification of the appropriate level for tests and full reporting of outcomes
☒	☐	Estimates of effect sizes (e.g. Cohen's *d*, Pearson's *r*), indicating how they were calculated

Our web collection on statistics for biologists contains articles on many of the points above.

Software and code

Policy information about availability of computer code

Data collection: No software was used.

Data analysis: The transmission dynamics mathematical model is written in C++ and R and model code can be found here: https://github.com/SwissTPH/openmalaria (v44), here: https://github.com/SwissTPH/r-openMalariaUtilities (v 23.2) and here: https://github.com/SwissTPH/AnophelesModel (v1.1.0). Data manipulation code, input parameters and processing code in R are available here: https://github.com/SwissTPH/ITNcascades.

Other packages and software used are:
Stan (version 2.32.2) using the Rstan interface (https://cran.r-project.org/web/packages/rstan, version 2.32.7)
R package chirps (https://cran.r-project.org/web/packages/chirps , v 0.1.4)

For manuscripts utilizing custom algorithms or software that are central to the research but not yet described in published literature, software must be made available to editors and reviewers. We strongly encourage code deposition in a community repository (e.g. GitHub). See the Nature Portfolio guidelines for submitting code & software for further information.

Data

Policy information about availability of data

All manuscripts must include a data availability statement. This statement should provide the following information, where applicable:

- Accession codes, unique identifiers, or web links for publicly available datasets
- A description of any restrictions on data availability
- For clinical datasets or third party data, please ensure that the statement adheres to our policy

All formatted datasets are available together with the code at https://github.com/SwissTPH/ITNcascades.

Research involving human participants, their data, or biological material

Policy information about studies with human participants or human data. See also policy information about sex, gender (identity/presentation), and sexual orientation and race, ethnicity and racism.

Reporting on sex and gender	Only male volunteers were recruited to sleep in the experimental huts based on informed consent.
Reporting on race, ethnicity, or other socially relevant groupings	The volunteers were recruited in the same village where the experimental huts are located. To account for differences in the attraction among the individuals, the variable for volunteers was adjusted for as a fixed effect in the models.
Population characteristics	The volunteers were adult aged 18 years old - 40 years old.
Recruitment	Participants were voluntarily recruited among the villagers living close to the experimental huts after written informed consent is obtained. The informed consent explained the risk and benefits of participating in the study, and right to withdraw and right to not to participate.
Ethics oversight	Ifakara Health Institute-Institutional Review Board (IHI-IRB), and National Institute for Medical Research Tanzania (NIMR)

Note that full information on the approval of the study protocol must also be provided in the manuscript.

Field-specific reporting

Please select the one below that is the best fit for your research. If you are not sure, read the appropriate sections before making your selection.

☐ Life sciences ☐ Behavioural & social sciences ☒ Ecological, evolutionary & environmental sciences

For a reference copy of the document with all sections, see nature.com/documents/nr-reporting-summary-flat.pdf

Life sciences study design

All studies must disclose on these points even when the disclosure is negative.

Sample size	*Describe how sample size was determined, detailing any statistical methods used to predetermine sample size OR if no sample-size calculation was performed, describe how sample sizes were chosen and provide a rationale for why these sample sizes are sufficient.*
Data exclusions	*Describe any data exclusions. If no data were excluded from the analyses, state so OR if data were excluded, describe the exclusions and the rationale behind them, indicating whether exclusion criteria were pre-established.*
Replication	*Describe the measures taken to verify the reproducibility of the experimental findings. If all attempts at replication were successful, confirm this OR if there are any findings that were not replicated or cannot be reproduced, note this and describe why.*
Randomization	*Describe how samples/organisms/participants were allocated into experimental groups. If allocation was not random, describe how covariates were controlled OR if this is not relevant to your study, explain why.*
Blinding	*Describe whether the investigators were blinded to group allocation during data collection and/or analysis. If blinding was not possible, describe why OR explain why blinding was not relevant to your study.*

Behavioural & social sciences study design

All studies must disclose on these points even when the disclosure is negative.

Study description	*Briefly describe the study type including whether data are quantitative, qualitative, or mixed-methods (e.g. qualitative cross-sectional, quantitative experimental, mixed-methods case study).*
Research sample	*State the research sample (e.g. Harvard university undergraduates, villagers in rural India) and provide relevant demographic*

Research sample	*information (e.g. age, sex) and indicate whether the sample is representative. Provide a rationale for the study sample chosen. For studies involving existing datasets, please describe the dataset and source.*
Sampling strategy	*Describe the sampling procedure (e.g. random, snowball, stratified, convenience). Describe the statistical methods that were used to predetermine sample size OR if no sample-size calculation was performed, describe how sample sizes were chosen and provide a rationale for why these sample sizes are sufficient. For qualitative data, please indicate whether data saturation was considered, and what criteria were used to decide that no further sampling was needed.*
Data collection	*Provide details about the data collection procedure, including the instruments or devices used to record the data (e.g. pen and paper, computer, eye tracker, video or audio equipment) whether anyone was present besides the participant(s) and the researcher, and whether the researcher was blind to experimental condition and/or the study hypothesis during data collection.*
Timing	*Indicate the start and stop dates of data collection. If there is a gap between collection periods, state the dates for each sample cohort.*
Data exclusions	*If no data were excluded from the analyses, state so OR if data were excluded, provide the exact number of exclusions and the rationale behind them, indicating whether exclusion criteria were pre-established.*
Non-participation	*State how many participants dropped out/declined participation and the reason(s) given OR provide response rate OR state that no participants dropped out/declined participation.*
Randomization	*If participants were not allocated into experimental groups, state so OR describe how participants were allocated to groups, and if allocation was not random, describe how covariates were controlled.*

Ecological, evolutionary & environmental sciences study design

All studies must disclose on these points even when the disclosure is negative.

Study description	Eight studies were assessed in this paper: six published (Kibondo, Odufuwa, Assenga, Martin, N'Guessan, Sovegnon) and two unpublished (BIT055, and BIT080). Only unpublished studies are commented here, and the reader is referred to the original publications for the other studies. The studies utilised Balanced Latin square design. Pyrethroid Insecticide treated nets treated with either Piperonyl Butoxide or chlorfenapyr were compared to pyrethroid treated ITNs only.
Research sample	Comparison of treated nets were done against free-flying wild Anopheles arabiensis in the experimental huts in Tanzania.
Sampling strategy	Sample size for each study was done in R software. A 1000 simulations was performed using estimates (mosquito mortality, and overall study variation) from previous studies for a non-inferiority study as recommended by the World Health Organization.
Data collection	Mosquitoes were collected using syphons and prokopacks, and held in paper cups by volunteers who slept in the hut. The cups were handed over to trained technicians who sorted the mosquitoes by species (An. arabiensis, An. funestus, and culex quinquefasciatus) morphologically, location (inside net, exit traps and inside hut) and status (unfed-dead, unfed-alive, fed-dead and unfed-alive). The mosquitoes were held in temperature-controlled environment with access to sugar to record delayed mortality at 24 or 72 hours.
Timing and spatial scale	The two studies were done at different time and all experiments were conducted nightly between 7 PM - 6 AM.<br>BIT055 (Nov-December 2020) was a 12 arm trial in 24 huts, each net condition was allocated at random to two huts each night and at the end of 36 nights each net condition was tested 72 times, with one night of break after every 6 nights of data collection.<br>BIT080 (April-May, 2023) was a 7 arm trial in 14 huts, each net condition was allocated at random to two huts each night and at the end of 49 nights each net condition was tested 98 times, with one night of break after every 7 nights of data collection.
Data exclusions	In each study where PBO nets was tested, the PBO ITN that is not Olyset Plus ITN was excluded, and in those where chlorfenapyr was tested, the chlorfenapyr net that is not Interceptor G2 was removed, given that the study is planned to assess the efficacy for Olyset Plus and Interceptor G2 being the first-in-class products.
Reproducibility	The studies were implemented following procedures required by the World Health Organization ITN guidance
Randomization	After initial random allocation, nets were rotated sequentially at the end of each round (after airing) to minimise error in treatment allocation. Sleepers rotated sequentially among huts each night of the study using a pre-prepared roster
Blinding	All nets were double size, and identifiable by a 4 digit code so that the participants and investigators were blinded to treatment allocation except the control.

Did the study involve field work? ☒ Yes ☐ No

Field work, collection and transport

Field conditions	Temperature and relative humidity of the holding room (where mosquitoes were held to assess delayed mortality) were controlled using air conditioner, bowls filled with water and dehumidifier to regulate the temperature to 22-32 degree centigrade and relative humidity to 60-100%.<br>The storage where bednets were kept during testing were kept at 25-32 degrees centigrade

Location	The study was conducted in Lupiro village (8.385oS and 36.670oE) in Ulanga District, south-eastern Tanzania adjacent to perennially swampy rice fields.
Access & import/export	Clearances from IHI-IRB and NIMR Tanzania were obtained respectively: IHI/IRB/No: 29-2020 and NIMR/HQ/R.8a/Vol.IX/3521 for BIT055 and, IHI/IRB/No: 35-2021 and NIMR/HQ/R.8a/Vol.IX/3957 for BIT080. For Assenga et al. 2025, ethical approval was and IHI/IRB/No: 21-2023 and NIMR/HQ/R.8a/Vol.IX/4558 for Tanzania and Ref 081-22/MSHPCMU/CNESVS-km for Côte d'Ivoire.
Disturbance	No disturbance was observed during the study.

Reporting for specific materials, systems and methods

We require information from authors about some types of materials, experimental systems and methods used in many studies. Here, indicate whether each material, system or method listed is relevant to your study. If you are not sure if a list item applies to your research, read the appropriate section before selecting a response.

Materials & experimental systems

n/a	Involved in the study
☒	☐ Antibodies
☒	☐ Eukaryotic cell lines
☒	☐ Palaeontology and archaeology
☐	☒ Animals and other organisms
☒	☐ Clinical data
☒	☐ Dual use research of concern
☒	☐ Plants

Methods

n/a	Involved in the study
☒	☐ ChIP-seq
☒	☐ Flow cytometry
☒	☐ MRI-based neuroimaging

Antibodies

Antibodies used	*Describe all antibodies used in the study; as applicable, provide supplier name, catalog number, clone name, and lot number.*
Validation	*Describe the validation of each primary antibody for the species and application, noting any validation statements on the manufacturer's website, relevant citations, antibody profiles in online databases, or data provided in the manuscript.*

Eukaryotic cell lines

Policy information about cell lines and Sex and Gender in Research

Cell line source(s)	*State the source of each cell line used and the sex of all primary cell lines and cells derived from human participants or vertebrate models.*
Authentication	*Describe the authentication procedures for each cell line used OR declare that none of the cell lines used were authenticated.*
Mycoplasma contamination	*Confirm that all cell lines tested negative for mycoplasma contamination OR describe the results of the testing for mycoplasma contamination OR declare that the cell lines were not tested for mycoplasma contamination.*
Commonly misidentified lines (See ICLAC register)	*Name any commonly misidentified cell lines used in the study and provide a rationale for their use.*

Palaeontology and Archaeology

Specimen provenance	*Provide provenance information for specimens and describe permits that were obtained for the work (including the name of the issuing authority, the date of issue, and any identifying information). Permits should encompass collection and, where applicable, export.*
Specimen deposition	*Indicate where the specimens have been deposited to permit free access by other researchers.*
Dating methods	*If new dates are provided, describe how they were obtained (e.g. collection, storage, sample pretreatment and measurement), where they were obtained (i.e. lab name), the calibration program and the protocol for quality assurance OR state that no new dates are provided.*

☐ Tick this box to confirm that the raw and calibrated dates are available in the paper or in Supplementary Information.

Ethics oversight	*Identify the organization(s) that approved or provided guidance on the study protocol, OR state that no ethical approval or guidance was required and explain why not.*

Note that full information on the approval of the study protocol must also be provided in the manuscript.

Animals and other research organisms

Policy information about studies involving animals; ARRIVE guidelines recommended for reporting animal research, and Sex and Gender in Research

Laboratory animals	Female pyrethroid-susceptible Anopheles gambiae (Ifakara strain) and resistant An. arabiensis (Kingani strain) were tested against insecticide treated nets using cone and tunnel bioassays following the WHO guidelines. Alive mosquitoes after exposure to insecticide were stored in the fridge (<4-8 degrees centigrade) to die before they were disposed.
Wild animals	Wild Anopheles and Culex mosquitoes captured in the experimental huts were assessed against the insecticide treated nets. After the study, alive mosquitoes were stored in the fridge (4-8 degree centigrade) to die before they were disposed.
Reporting on sex	Female mosquitoes were considered and used to assess the efficacy of the insecticide products given that it is the female mosquitoes that transmit the diseases. The identification was done morphologically by trained medical entomologist
Field-collected samples	The mosquitoes collected in the experimental huts were from the rice farms located adjacent to the experimental huts. After collection, the mosquitoes were transferred to the environmentally controlled rooms for sorting and identification morphologically, and a few of the dead Anopheles mosquitoes were transferred in silicon jells to the lab for sibling identification molecularly.
Ethics oversight	Clearances from IHI-IRB and NIMR Tanzania were obtained respectively IHI/IRB/No: 29-2020 and NIMR/HQ/R.8a/Vol.IX/3521 for BIT055, IHI/IRB/No: 35-2021 and NIMR/HQ/R.8a/Vol.IX/3957 for BIT080. For Assenga et al., ethical approval was and IHI/IRB/No: 21-2023 and NIMR/HQ/R.8a/Vol.IX/4558 for Tanzania and Ref 081-22/MSHPCMU/CNESVS-km for Côte d'Ivoire.

Note that full information on the approval of the study protocol must also be provided in the manuscript.

Clinical data

Policy information about clinical studies

All manuscripts should comply with the ICMJE guidelines for publication of clinical research and a completed CONSORT checklist must be included with all submissions.

Clinical trial registration	*Provide the trial registration number from ClinicalTrials.gov or an equivalent agency.*
Study protocol	*Note where the full trial protocol can be accessed OR if not available, explain why.*
Data collection	*Describe the settings and locales of data collection, noting the time periods of recruitment and data collection.*
Outcomes	*Describe how you pre-defined primary and secondary outcome measures and how you assessed these measures.*

Dual use research of concern

Policy information about dual use research of concern

Hazards

Could the accidental, deliberate or reckless misuse of agents or technologies generated in the work, or the application of information presented in the manuscript, pose a threat to:

No	Yes	
☐	☐	Public health
☐	☐	National security
☐	☐	Crops and/or livestock
☐	☐	Ecosystems
☐	☐	Any other significant area

Experiments of concern

Does the work involve any of these experiments of concern:

No	Yes	
☐	☐	Demonstrate how to render a vaccine ineffective
☐	☐	Confer resistance to therapeutically useful antibiotics or antiviral agents
☐	☐	Enhance the virulence of a pathogen or render a nonpathogen virulent
☐	☐	Increase transmissibility of a pathogen
☐	☐	Alter the host range of a pathogen
☐	☐	Enable evasion of diagnostic/detection modalities
☐	☐	Enable the weaponization of a biological agent or toxin
☐	☐	Any other potentially harmful combination of experiments and agents

Plants

Seed stocks	N/a
Novel plant genotypes	N/a
Authentication	N/a

ChIP-seq

Data deposition

☐ Confirm that both raw and final processed data have been deposited in a public database such as GEO.

☐ Confirm that you have deposited or provided access to graph files (e.g. BED files) for the called peaks.

Data access links<br>*May remain private before publication.*	*For "Initial submission" or "Revised version" documents, provide reviewer access links. For your "Final submission" document, provide a link to the deposited data.*
Files in database submission	*Provide a list of all files available in the database submission.*
Genome browser session<br>(e.g. UCSC)	*Provide a link to an anonymized genome browser session for "Initial submission" and "Revised version" documents only, to enable peer review. Write "no longer applicable" for "Final submission" documents.*

Methodology

Replicates	*Describe the experimental replicates, specifying number, type and replicate agreement.*
Sequencing depth	*Describe the sequencing depth for each experiment, providing the total number of reads, uniquely mapped reads, length of reads and whether they were paired- or single-end.*
Antibodies	*Describe the antibodies used for the ChIP-seq experiments; as applicable, provide supplier name, catalog number, clone name, and lot number.*
Peak calling parameters	*Specify the command line program and parameters used for read mapping and peak calling, including the ChIP, control and index files used.*
Data quality	*Describe the methods used to ensure data quality in full detail, including how many peaks are at FDR 5% and above 5-fold enrichment.*
Software	*Describe the software used to collect and analyze the ChIP-seq data. For custom code that has been deposited into a community repository, provide accession details.*

Flow Cytometry

Plots

Confirm that:

- [ ] The axis labels state the marker and fluorochrome used (e.g. CD4-FITC).
- [ ] The axis scales are clearly visible. Include numbers along axes only for bottom left plot of group (a 'group' is an analysis of identical markers).
- [ ] All plots are contour plots with outliers or pseudocolor plots.
- [ ] A numerical value for number of cells or percentage (with statistics) is provided.

Methodology

Sample preparation	*Describe the sample preparation, detailing the biological source of the cells and any tissue processing steps used.*
Instrument	*Identify the instrument used for data collection, specifying make and model number.*
Software	*Describe the software used to collect and analyze the flow cytometry data. For custom code that has been deposited into a community repository, provide accession details.*
Cell population abundance	*Describe the abundance of the relevant cell populations within post-sort fractions, providing details on the purity of the samples and how it was determined.*
Gating strategy	*Describe the gating strategy used for all relevant experiments, specifying the preliminary FSC/SSC gates of the starting cell population, indicating where boundaries between "positive" and "negative" staining cell populations are defined.*

- [ ] Tick this box to confirm that a figure exemplifying the gating strategy is provided in the Supplementary Information.

Magnetic resonance imaging

Experimental design

Design type	*Indicate task or resting state; event-related or block design.*
Design specifications	*Specify the number of blocks, trials or experimental units per session and/or subject, and specify the length of each trial or block (if trials are blocked) and interval between trials.*
Behavioral performance measures	*State number and/or type of variables recorded (e.g. correct button press, response time) and what statistics were used to establish that the subjects were performing the task as expected (e.g. mean, range, and/or standard deviation across subjects).*

Acquisition

Imaging type(s)	*Specify: functional, structural, diffusion, perfusion.*
Field strength	*Specify in Tesla*
Sequence & imaging parameters	*Specify the pulse sequence type (gradient echo, spin echo, etc.), imaging type (EPI, spiral, etc.), field of view, matrix size, slice thickness, orientation and TE/TR/flip angle.*
Area of acquisition	*State whether a whole brain scan was used OR define the area of acquisition, describing how the region was determined.*

Diffusion MRI ☐ Used ☐ Not used

Preprocessing

Preprocessing software	*Provide detail on software version and revision number and on specific parameters (model/functions, brain extraction, segmentation, smoothing kernel size, etc.).*
Normalization	*If data were normalized/standardized, describe the approach(es): specify linear or non-linear and define image types used for transformation OR indicate that data were not normalized and explain rationale for lack of normalization.*
Normalization template	*Describe the template used for normalization/transformation, specifying subject space or group standardized space (e.g. original Talairach, MNI305, ICBM152) OR indicate that the data were not normalized.*
Noise and artifact removal	*Describe your procedure(s) for artifact and structured noise removal, specifying motion parameters, tissue signals and physiological signals (heart rate, respiration).*

Volume censoring

Define your software and/or method and criteria for volume censoring, and state the extent of such censoring.

Statistical modeling & inference

Model type and settings

Specify type (mass univariate, multivariate, RSA, predictive, etc.) and describe essential details of the model at the first and second levels (e.g. fixed, random or mixed effects; drift or auto-correlation).

Effect(s) tested

Define precise effect in terms of the task or stimulus conditions instead of psychological concepts and indicate whether ANOVA or factorial designs were used.

Specify type of analysis: ☐ Whole brain ☐ ROI-based ☐ Both

Statistic type for inference

(See Eklund et al. 2016)

Specify voxel-wise or cluster-wise and report all relevant parameters for cluster-wise methods.

Correction

Describe the type of correction and how it is obtained for multiple comparisons (e.g. FWE, FDR, permutation or Monte Carlo).

Models & analysis

n/a	Involved in the study
☐	☐ Functional and/or effective connectivity
☐	☐ Graph analysis
☐	☐ Multivariate modeling or predictive analysis

Functional and/or effective connectivity

Report the measures of dependence used and the model details (e.g. Pearson correlation, partial correlation, mutual information).

Graph analysis

Report the dependent variable and connectivity measure, specifying weighted graph or binarized graph, subject- or group-level, and the global and/or node summaries used (e.g. clustering coefficient, efficiency, etc.).

Multivariate modeling and predictive analysis

Specify independent variables, features extraction and dimension reduction, model, training and evaluation metrics.
